# Covalent Organic Frameworks for Chemical and Biological Sensing

**DOI:** 10.3390/molecules27082586

**Published:** 2022-04-18

**Authors:** Shiji Zhang, Danqing Liu, Guangtong Wang

**Affiliations:** 1 School of Material Science and Chemical Engineering, Harbin University of Science and Technology, Harbin 150080, China; zhangcentury975@gmail.com; 2 Key Laboratory of Micro-Systems and Micro-Structures Manufacturing (Ministry of Education), Harbin Institute of Technology, Harbin 150080, China

**Keywords:** covalent organic frameworks, porous material, sensor, gas, humidity, ions, explosives, drug, biosensing

## Abstract

Covalent organic frameworks (COFs) are a class of crystalline porous organic polymers with polygonal porosity and highly ordered structures. The most prominent feature of the COFs is their excellent crystallinity and highly ordered modifiable one-dimensional pores. Since the first report of them in 2005, COFs with various structures were successfully synthesized and their applications in a wide range of fields including gas storage, pollution removal, catalysis, and optoelectronics explored. In the meantime, COFs also exhibited good performance in chemical and biological sensing, because their highly ordered modifiable pores allowed the selective adsorption of the analytes, and the interaction between the analytes and the COFs’ skeletons may lead to a detectable change in the optical or electrical properties of the COFs. In this review, we firstly demonstrate the basic principles of COFs-based chemical and biological sensing, then briefly summarize the applications of COFs in sensing some substances of practical value, including some gases, ions, organic compounds, and biomolecules. Finally, we discuss the trends and the challenges of COFs-based chemical and biological sensing.

## 1. Introduction

Detecting chemicals or biomolecules is one of the essential goals of analytical chemistry and plays an important role in a wide range of fields including environmental protection, clinic diagnosis, health monitoring, public security, food industry, et al. Thanks to the fast development of instrument science and material science in recent decades, instruments for detecting diverse analytes have been developed. Nowadays, various spectrophotometers, chromatographs, and electrochemical analyzers can be found as conventional analytical equipment in laboratories, factories, and even hospitals. A large number of chemicals and biomolecules can be detected with satisfactory accuracy. However, due to the growing concern for environmental security and healthcare, the demand for fast, real-time, and portable analytical instruments has been increasing. In recent years, plenty of efforts have been devoted to developing portable, wearable, or even implantable sensors for detecting impurities, pollutants, drugs, and physiological indexes [1,2,3,4,5,6,7].

In principle, two key concerns should be carefully considered for detecting a particular analyte. One is to selectively recognize and separate the analyte from the samples. Although some analytes with special physical properties can be separated by simple methods such as filtration, centrifugation, evaporation, et al., most analytes’ separation so far relies on molecular recognition and selective adsorption based on physical, chemical, or biological interaction. Therefore, it is crucial to design and prepare a sort of material that can selectively interact and capture the analyte from the samples during the development of a chemical or biological sensor. Until now, 2D materials such as graphene [8,9], BN [10], MoS_2_ [11], WS_2_ [12], and MXene [13], and porous materials including zeolites [14], metal organic frameworks (MOFs) [15,16,17], and covalent organic frameworks (COFs) [18,19,20] were usually chosen as the candidates for selective analytes’ capture. A number of chemical or biological sensors based on the above materials have been constructed. The other key concern for chemical and biological sensing is converting the uptake of the analyte to a detectable signal. The signal can be optical, electrical, thermal, or acoustic. In practice, electrical and optical signals were usually applied since they can be real-time and quantitatively monitored. 

**Figure 1:**
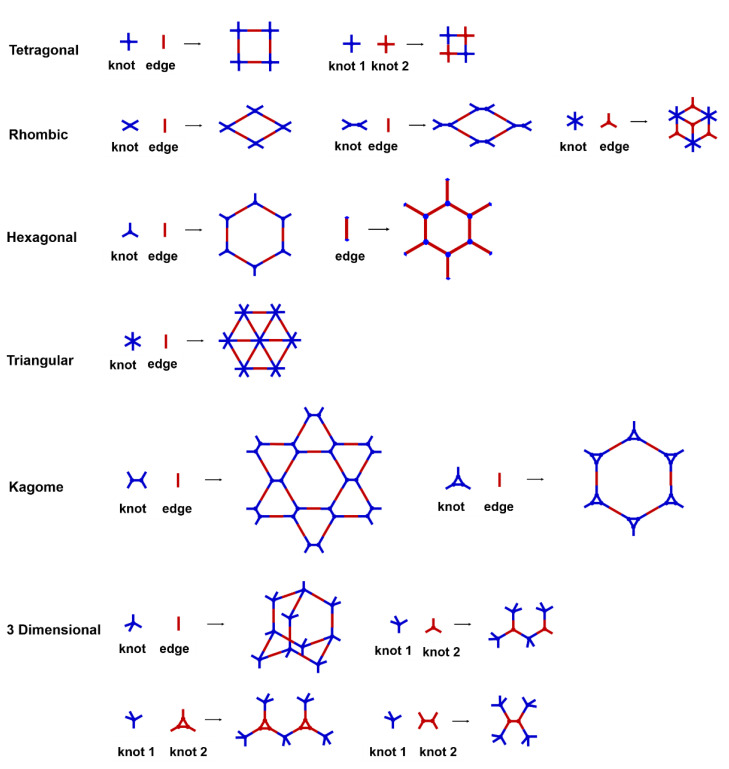
The basic topology of the COFs.

COFs are a class of crystalline porous organic polymers with polygonal porosity and highly ordered structures [21,22,23]. As shown in Figure 1, the porosity of the COFs originated from their unique structural design of monomers. In general, the monomers for synthesizing COFs can be roughly divided into two categories. One is the star-like multi-functional monomer that acts as the “knot” in the framework and the other is the linear double functional monomer that can be used as the “edge”. The knots and edges are usually linked by dynamic covalent bonds such as imine, borate ester, hydrazone, carbon-carbon double bond, et al. (Table 1). The dynamic nature of these linkages allows the formation of highly regular, repeated structures with ordered polygonal pores of which the sizes and shapes are determined by the molecular structures of the “knot” and “edge” molecules [19,21,24,25]. Furthermore, functional substituents can be easily introduced to the pore of the COFs by pre-linking it onto the “knots” or “edges” before the synthesis of the COFs or by immobilizing it onto the wall of the pore after the formation of the COFs. In addition, the pores and functional groups on the COFs’ skeletons allows further modification of the COFs by loading nanoparticles or immobilizing nucleic acid or enzymes into their pores or onto their 2D sheets [26,27,28,29]. Thus, it seems that there are infinite possibilities for creating new functional COFs or COF-based hybrid materials. Since the first report by Yaghi et al., in 2005 [30], numerous COFs with various structures and functions have been presented. The application of COFs has been extended to the fields of energy storage and conversion [31,32], optoelectronics [33], gas adsorption and separation [31,34,35,36], sensing [18,19,20], catalysis [37,38,39,40,41], pollutant removal [42,43], battery [44], drug release [45], and so on. 

COFs nowadays have been proved to be an ideal platform for chemical and biological sensing. The ordered pore structure of COFs benefits the selective adsorption of the analytes. More importantly, by the pre-design of the monomers, binding sites that can specifically interact with the analyte can be introduced into the “knots” or “edges” of the COFs to further enhance the selectivity of the analytes’ adsorption. Therefore, COFs have exhibited a great advantage in the selective capture and enrichment of the analyte. Furthermore, the analyte adsorption may also affect the optical, electrical, or catalytic properties of the COFs, resulting in a detectable change of color, fluorescence, conductivity, capacitance, or (photo) electrochemical currents, et al. [18]. It can be found that the two key problems of fabricating a chemical or biological sensor are possibly simultaneously solved by one well-designed COF or COF-based hybrid material. Therefore, plenty of chemical or biological sensors on the basis of COFs or COFs-based hybrids have been reported in recent years [18,19,20,21,22,46].

In this review, we firstly discuss the basic principles to design a COF that can selectively adsorb the analyte, then demonstrate the detectable signals that are probably generated by COFs resulting from the analyte adsorption. Successively, we briefly summarize the application of COFs in detecting some substances of practical value, including some gases, ions, organic compounds, and biomolecules. Finally, we discuss the main challenges and future perspectives of COFs-based chemical or biological sensors.

## 2. Basic Principles of COF-Based Sensing 

In recent years, COF-based chemical or biological sensors on the basis of various mechanisms were presented. Although there are still a few exceptions, some principles, strategies, and experiences for fabricating a COF-based sensor can be summarized from the numerous reports. In this section, we will firstly discuss the basic principles for designing a COF that can enrich the analyte. Then we demonstrate some detectable signals that can be produced by the adsorption of the analyte. 

### 2.1. Designing a COF or COF-Based Hybird Material for Selective Adsorption

**Figure 2:**
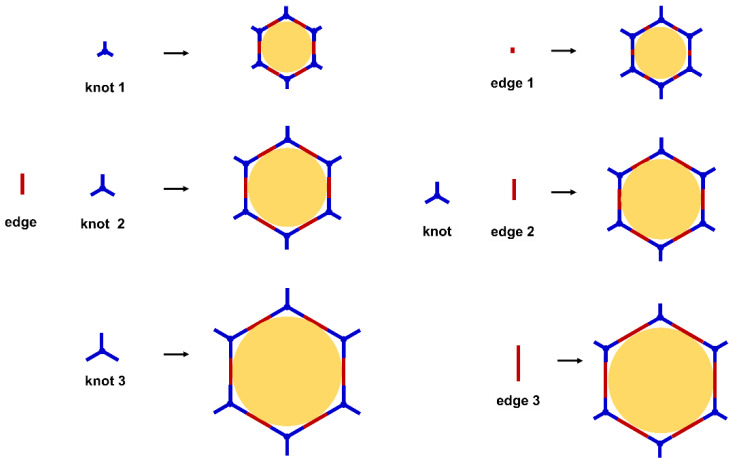
Modulating the pore size of the COFs.

**Figure 3:**
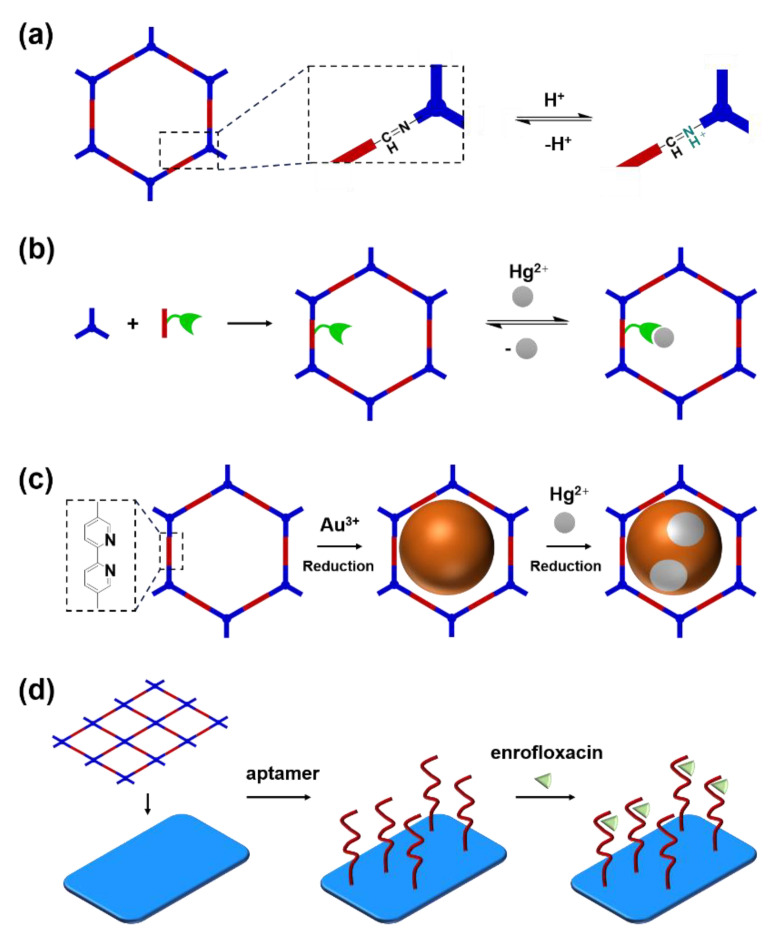
Introducing the binding site that can selectively capture the analyte. (**a**) capturing H^+^ by imine linkage; (**b**) Intruding thioether binding site for capturing Hg^2+^; (**c**) Introducing AuNPs by in situ growth for mercury capture; (**d**) Externally immobilizing enrofloxacin-targeted aptamer onto the 2D sheet of the COF for capturing enrofloxacin.

The porous structure of the COF results in its extraordinary adsorption capacity. However, compared with other types of porous materials, the predominant characteristic of COFs is the designability of the pores. Inspired by the recognition between the enzyme and its substrate, the key points to design a COF that can enrich the analyte are creating a suitable space allowing the load of the analyte and introducing binding sites to selectively interact with the analyte. Hence, the shape and size of the COF pores should be firstly considered. In general, the shape of the pores is mainly determined by the topology of the “knot” monomer (Figure 1). For example, to obtain a hexagonal pore, three-armed planar rigid “knot” monomers, such as the derivatives of triazine, triphenyl benzene, triphenylene derivatives, et al. were usually applied. In addition, four-armed planar rigid “knot” monomers were often used for fabricating a tetragonal pore, while the size of the COF pores depends on the length of both the “edge” monomer and the arms of the “knot” monomers. As shown in Figure 2, it can be easily enlarged by choosing a longer “edge” monomer or a “knot” molecule with longer arms. Thus, it can be found that both the shape and size of the COFs pores can be pre-designed by choosing the “knot” and “edge” monomers [21,24]. To effectively adsorb the analyte, the pore shape and size should be carefully designed to at least allow the analyte molecule to enter. Furthermore, for discriminating the interferents of a similar molecular shape and size, in general, a binding site is needed that can selectively interact with the analyte. The binding site can be the innate groups on the COF skeleton. For example, as shown in Figure 3a, the imine linkage is pH sensitive. It is a good binding site for capturing H^+^ in the solution or the acid gases like HCl and Trifluoroacetic acid (TFA) [47,48]. However, in other cases, as shown in Figure 3b, the binding site is pre-linked onto one of the monomers before the synthesis of the COFs. For example, thiol or thioether groups are able to selectively adsorb the heavy metal ions like Hg^2+^. Thus, thioether was covalently pre-linked onto the “edge” monomer, yielding a thioether-containing COF (COF−LZU8) [42]. It was shown that COF−LZU8 performed well in selectively capturing and detecting Hg^2+^. In addition, the binding site can be also introduced after the formation of COFs, which is also called “post-functionalization” of COFs. For instance, Au nanoparticles (AuNPs) can form an amalgam layer with Hg^2+^ under the reduction of citric acid (Figure 3c). Hence, to efficiently capture and detect the trace amount of Hg^2+^, AuNPs were doped on the nanosheet of a bipyridine-containing COF by the in situ growth method to form a COF-based hybrid material. It was found that the huge specific surface area of the COF-AuNPs hybrid material can significantly enhance the sensitivity of the Hg^2+^ detection [29]. Moreover, except for in situ growth, the binding site can be also externally introduced onto the 2D sheet of the COFs. For example, as shown in Figure 3d, to selectively detect enrofloxacin, a kind of antibiotic, an enrofloxacin-targeted aptamer was immobilized onto a 2D sheet of COF via π-π stacking and electrostatic interaction to yield an aptamer-functionalized COF hybrid [49]. Then the hybrid was coated onto a gold electrode. It can selectively capture the enrofloxacin in the sample solution and result in a detectable change in the electrode conductivity. 

### 2.2. Signals Produced by the Adsorption of the Analyte

Apart from the selective uptake and enrichment of the analyte, the other key problem in constructing a COF-based sensor is to make the uptake of the analyte produce a detectable signal. By ingenious structural design, a large number of COFs of which the color, fluorescence, conductivity or capacitance can quantitatively change with the uptake of the analyte have been synthesized [18]. Besides that, in some reports the adsorption of the analyte can also produce some detectable signals which do not originate from COFs [29,50]. In this section, we will briefly discuss the generation, application, and advantages of the above signals in COF-based sensors. In addition, other analytical techniques such as QCM, Raman spectra, MS, et al. were also used occasionally. These techniques will be discussed with the particular examples demonstrated in the next section. 

#### 2.2.1. Fluorescence 

Among the detectable signals caused by the uptake of the analyte, fluorescence change of COFs including fluorescence enhancement (“turn on”) and fluorescence quenching (“turn off”) is the most frequently used to respond to the concentration of analyte. Due to the rigid structure, the large π-conjugated fluorescent chromophores, such as phenyl, naphthalenyl, pyrenyl, perylenyl, triazine, and triphyenyl-benzene are usually applied for building COFs [51,52]. Moreover, the conjugated linkages like imine and olefins can further enlarge the π-conjugated structure in the COFs. Therefore, it is not unusual to find a COF with fluorescent emission. However, it was found that the adsorption of some analytes may influence the fluorescent emission, including the change of the intensity and movement of the emission peak. The fluorescence change may be induced by various mechanisms, such as the charge and energy transfer between the COF and analyte, aggregation-induced emission, and exciplex formation, et al. The most common one among the above mechanisms is the charge transfer from an electron-rich COF to an electron-deficient analyte. It can lead to a significant fluorescent quenching which is relative to the concentration of the analyte. In the following demonstration, it can be found that many COF-based nitroaromatic explosive sensors, iodine sensors, and transition metal ions sensors are constructed by the charge transfer mechanism.

#### 2.2.2. Chromism

Chromism is a signal that can be directly found by naked eyes. The advantage of the sensing approach based on chromism is easy-operate. It is very convenient to be further developed into rapid testing equipment like test paper, test kit, and wearable monitoring devices. Therefore, the COF-based sensors based on chromism are frequently reported. The color change of the COFs can be induced in different manners. Firstly, the non-covalent interaction between COFs and the analyte disturbs the electronic transition responsible for the coloration. For example, the adsorption of molecules with different polarities can influence the intramolecular charge transfer from the electron donor to the electron receptor on the COF skeleton, causing a red or blue shift of the UV-Vis absorbance [53]. Secondly, in some COF-based chromism sensors, the interaction with the analyte can isomerize some groups on the COF skeleton, resulting in the color change [54,55]. In addition, it should be noted that there is also a type of COF-based chromism sensor of which the color change is not caused by the COF itself. For example, in some sensors, COFs are used as a catalyst or a catalyst carrier, and the adsorption of the analyte can influence the catalytic capacity which can be detected by the formation of the colored product. Thus, the concentration of the analyte can be determined by the color change [29,50].

#### 2.2.3. Capacitance and Conductivity

The adsorption of the analyte can lead to a change in the capacitance and conductivity of the COFs [56,57,58]. Hence the concentration of the analyte can be also determined by the change of such two electrical signals. In general, the quantitative measurement of electrical signals is much more convenient compared with photo signals. The measuring devices involved are much simpler. Therefore, COF-containing sensors based on the change of capacitance and conductivity are more likely to be realized on a chip-like device. Many presented works have demonstrated that a fingertip-size interdigital electrode (IDE) coated with a specific COF can be applied for real-time detection of humidity, corrosive gases, or toxic gases since their adsorption results in the change of the capacitance or conductivity of the COFs. It can be expected that such a type of device can be further developed into portable, wearable, or even implantable sensors for real-time sensing.

#### 2.2.4. Electrochemical or Photoelectrochemical Signals

Electrochemical or photoelectrochemical signals are generated by the redox reaction of the active substance. Plenty of analytical techniques based on electrochemistry or photoelectrochemistry have been built. The signal produced by electrochemical or photoelectrochemical reactions can be influenced by many factors such as the concentration of the active substances, the conductivity of the electrode/electrolyte interface, the existence of catalysts, and so on. In recent years, a number of examples of electrochemical or photoelectrochemical analysis with the participation of COFs were reported. The COFs coated on the electrode in these examples can play various roles including: (1) enriching the analyte that can be directly detected by the electrochemical or photoelectrochemical method; (2) concentrating the analytes to inhibit or facilitate the transportation of charges or substances; (3) a carrier for immobilizing the catalyst which adsorption can be affected by the analytes. All of the above allow a significant influence of the analyte on the electrochemical or photoelectrochemical signal. Compared with the above type of COF-based sensors the advantage of the electrochemical or photoelectrochemical signal is the high sensitivity. It is not rare to realize trace amounts of the analyte at or lower than ppb or ng/mL level with this type of COF-based sensor.

## 3. Application of COFs for Sensing Various Analytes

### 3.1. Gas Sensing

Generally, the key challenge of gas sensing is to capture the target molecule from the sample. Therefore, porous materials like zeolite, MOFs, and COFs show a great advantage in gas sensing. Driven by the increasing demand for healthcare and environmental protection, some COFs that can be used for detecting irritating, harmful, or toxic gases have been developed (Table 2).

#### 3.1.1. Acidic and Alkaline Gases

**Figure 4:**
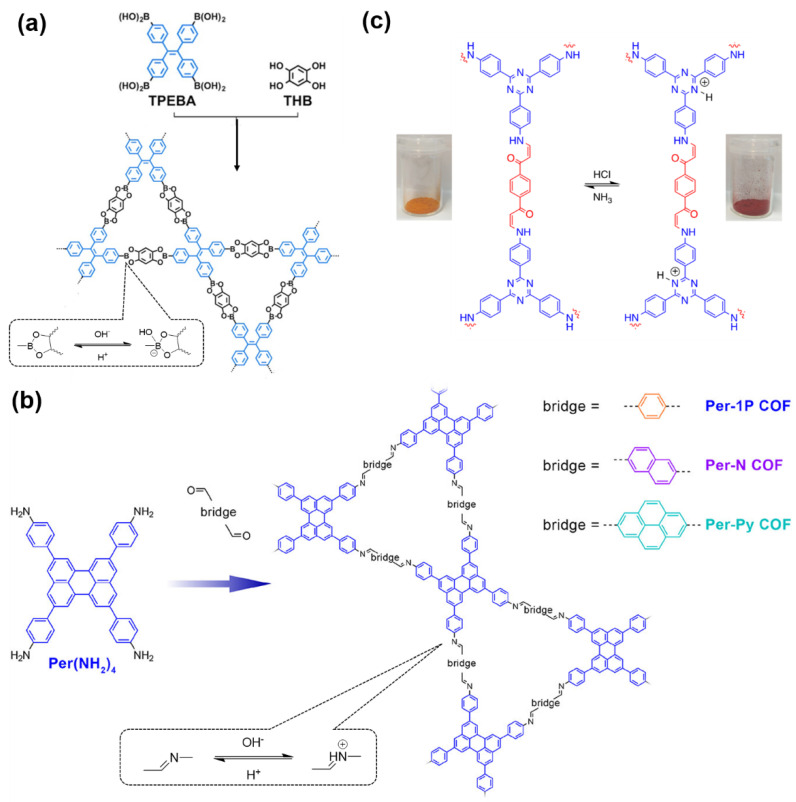
(**a**) COF for NH_3_ sensing based on the protonation and deprotonation of boronate linkage [60]. Copyright 2016 American Chemical Society; (**b**) COF for HCl sensing is based on the protonation and deprotonation of imine linkage [47]. Copyright 2019 American Chemical Society; (**c**) COF for HCl, NH_3_ sensing based on the protonation and deprotonation of imine linkage [57]. Copyright 2019 Nature Publishing Group.

Ammonia is widely applied in agriculture, the chemical industry, and refrigerated storage. Ammonia leakage can lead to discomfort and do harm to health. Although the pungent odor of ammonia can put us on high alert immediately, slow leakage of ammonia will decrease our sense of smell to find it. Therefore, it is necessary to detect trace amounts of ammonia. Early in 2010, O. M. Yaghi reported that the boronate linkage for synthesizing COFs can be used for ammonia uptake due to the Lewis acid–base interaction [59]. In 2016, D. Jiang and coworkers reported a highly emissive covalent organic framework (TPE−Ph−COF) based on dynamic boronate linkage [60] (Figure 4a). The boronate linkages can serve as a Lewis acid when it interacts with ammonia, which is a Lewis base. The interaction with ammonia significantly decreased the luminescence of TPE−Ph−COF. Hence, the dispersion of TPE−Ph−COF in organic solvent, such as toluene or cyclohexane, can be used for ammonia sensing. It can be found that the fluorescence of TPE−Ph−COF linearly decreased with the concentration of NH_3_ and 1 ppm NH_3_ could lead to a 30% reduction in fluorescence intensity. This result indicated that TPE−Ph−COF is a good candidate for detecting trace amounts of ammonia.

**Table 2:** COF-based gas sensing.

Analyte	Year	COF Names	Specific Binding Site	Type of Detectable Signal	Detection Range	LOD	Reference
NH_3_	2016	TPE−Ph−COF	Boronate	Fluorescence (turn off)	-	sub ppm level	[60]
	2018	HMP−TAPB−1HMP−TAPB−1	Heptazine	conductivity	1–200 ppm	1 ppm	[61]
	2018	COP−COP−1	Triazine	Fluorescence (turn on)	-	5.8925 × 10^−4^ mL/mL	[62]
	2019	Ph−An−COF	Boronate	Fluorescence (turn off)	-	-	[63]
	2019	COF−DC−8	-	Conductivity	2–80 ppm	56.8–70 ppb	[64]
	2021	TAPB−BPDA COF	Imine	Conductivity	5–100 ppm	10 ppb	[65]
TFA	2019	Per−N COF	Imine	Chromism	0.035–110 mg L^−1^	35 μg L^−1^	[47]
HCl	2018	COP−COP−1	Triazine	Fluorescence (turn off)		1.0967 × 10^−4^ mL/mL	[62]
	2019	PBHP−TAPT COF	Triazine	Chromism, conductivity	20–3000 ppm	20 ppm	[57]
	2019	COF−ETBA−DAB	Imine	Fluorescence		4.7 ppm	[66]
	2020	BCTB−BCTA COF	Imine	Fluorescence (turn off)	1–25 mM	10 nM	[48]
H_2_O	2013	TAPP−DHNDA−COF	Iminol	Chromism	20–100% RH		[54]
	2017	COF−TXDBA	Boronate	Conductivity	11–98% RH		[56]
	2018	Py−TT COF		Chromism	0.64–0.98 p/p_0_		[53]
	2020	TAPB−PDA−OH COF	Iminol	Chromism			[55]
	2021	DUT−175	Imine	Chromism	33–94% RH		[67]
Benzene	2020	BTA−TAPT-COF	Aromatic group	Capacitance	500 ppb–100 ppm	340 ppb	[58]
NO_2_	2019	COF−DC−8		Conductivity	2–40 ppm	1–16 ppb	[64]
	2020	CON−10		Conductivity		2.242 ppb	[68]
	2020	T−2DP		Conductivity	0.15–5 ppm	2.2 ppb	[69]
	2021	NiPc−CoTAA		Conductivity	1–40 ppm		[70]
NO	2019	COF−DC−8		Conductivity	0.02–40 ppm	1–5 ppb	[64]
H_2_S	2017	PNT−1	Triazine, pyridine	Fluorescence (turn off)		53 ppb	[71]
	2019	COF−DC−8		Conductivity	2–80 ppm	121 ppb	[64]
O_3_	2021	P−COFTPB−DMTP−COF	imine	Chromism		0.1 ppm	[72]

It can be found that the acid–base interaction between NH_3_ and the skeleton of the COFs played an essential role in NH_3_ detection since NH_3_ is a typical alkaline gas [60,61,62,63,64,65]. Correspondingly, it can be expected that acidic gas, such as HCl and TFA, can also be detected in a similar vein [47,48,57,62,66]. As with the boronate bond, the imine bond (Schiff base) is another frequently used dynamic linkage for COF synthesis. However, the imine linkage in COFs is usually a proton receptor that can easily react with acidic gases, allowing imine-based COFs to be sensitive to acids [47,66]. For example, F. Auras and co-workers developed a series of star-shaped COFs (Per-1P, Per−N, and Per-Py COFs) via the condensation of perylene tetraaniline with the phenylene-, naphthalene-, or pyrene-based dicarbaldehydes (Figure 3b) [47]. They found that the imine linkages of the star-shaped COFs can be reversibly protonated, resulting in a marked color change of the COFs. For example, the color of the Per−N-COFs thin film quickly changed from yellow to dark brown upon exposure to the vapor of trifluoroacetic acid (TFA). Its adsorption band around 385 nm decreased with the concentration of TFA vapor, simultaneously a new increasing absorption band around 450 nm could be observed. As a colorimetric acid vapor sensor, the detection limit of Per−N-COFs to trifluoroacetic acid can be as low as 35 μg L^−1^ and the response range was at least four orders of magnitude.

Apart from the imine bond, triazine is another acid-sensitive group that has been frequently used in the fabrication of covalent organic porous materials. The long pair of three N atoms in triazine is a good H^+^ acceptor. In 2018, N. Xu and coworkers prepared a high-performance fluorescent triazine-based covalent organic polymer (COP−COP−1) sensor for detecting both HCl and NH_3_ gases [62]. It was found that when the COP−COP−1 was exposed to gaseous HCl, a redshift of the fluorescence happened and the intensity of the fluorescence decreased linearly with increasing HCl concentration. Conversely, NH_3_ can lead to recovery of both color and fluorescence of the COP−COP−1 saturated by HCl, indicating that COP−COP−1 saturated by HCl can be applied as NH_3_ detection. Later, R. Kulkarni and coworkers reported a triazine-based COF (PBHP−TAPT COF) with similar property [57] (Figure 3c). Gaseous HCl can protonate the triazine groups in PBHP−TAPT COF and quickly turn its color from yellow to red. A very low concentration of HCl (20–50 ppm) can result in a detectable color change. The reverse color switch can be realized by the deprotonation by NH_3_. Furthermore, R. Kulkarni and coworkers found that the protonation of the triazine groups enhanced the conductivity of PBHP−TAPT COF by 170-fold, indicating that apart from the colorimetric method the acidic gas detection by PBHP−TAPT COF can be conducted by also monitoring electrical conductivity.

Notably, the COF-based detection of NH_3_ without acid–base interaction was also reported [65]. In 2021, F. Niu and coworkers synthesized a COF (TAPB−BPDA) via the condensation of 4,4′-biphenyldicarboxaldehyde with 1,3,5-tris(4-aminophenyl)benzene catalyzed by a proper amount of Sc(OTf)_3_ and coated it onto an IDE. It can be measured that the resistance of TAPB−BPDA decreased when it was exposed to NH_3_. The limit of detection (LOD) of this resistance-based approach can be as low as 10 ppb. Meanwhile, owing to the hydrogen bond between the COF and analyte, the NH_3_ sensing of TAPB−BPDA exhibited a good selectivity. Other gases, such as NO_2_, NO, SO_2_, H_2_S, H_2_, CO, CO_2_, et al., cannot result in the resistance decrease. The work by F. Niu and coworkers provide an example of NH_3_ sensing with noncovalent interaction.

#### 3.1.2. Water Vapor (Humidity) Sensing

Humidity is one of the essential indexes of our working and living environment. The equipment for real-time humidity monitoring can be widely found in laboratories and factories. In recent years, developing wearable respiratory monitoring devices has drawn much attention in the fields of healthcare and sports, because they can be used to record the breathing rate and depth of patients and athletes. For fabricating a wearable respiratory monitor, a sensitive humidity responsive material is vital. Due to the designable pore structure, COFs are good candidates for selectively capturing and detecting water vapor in the air [53,54,55,56,67].

**Figure 5:**
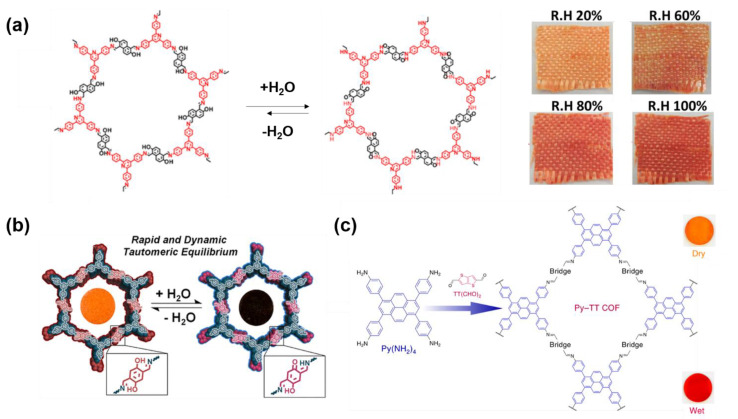
(**a**) TAPP−DHNDA−COF/aramid hybrid material for humidity detection [54]. Copyright 2013 American Chemical Society; (**b**) Py–TT COF thin film for humidity detection [53]. Copyright 2018 Nature Publishing Group; (**c**) Py−TT COF for humidity detection [55]. Copyright 2020 American Chemical Society.

The mechanism firstly used for humidity detection is the keto-iminol tautomerism. Early in 2013, X. H. Liu and coworkers solvothermally prepared crystalline COF (TAPP−DHNDA−COF) nanofibers by the imine condensation between 2,4,6-tris(4-aminophenyl) pyridine (TAPP) and 2,6-Dihydroxynaphthalene-1,5-dicarbaldehyde (DHNDA) [54]. SEM images showed that the nanofibers can form when the time of solvothermal synthesis was longer than 24 h. By using aramid fabric as a nucleating agent, X. H. Liu and coworkers successfully prepared a TAPP−DHNDA−COF/aramid hybrid material, where TAPP−DHNDA−COF nanofiber was coated on the surface of the aramid fibers. The hybrid material exhibited a reversible colorimetric humidity-sensitive property because the addition of H_2_O can disturb the equilibrium of keto-iminol tautomerism (Figure 5a). The color of the hybrid can switch from yellow to red gradually with the increasing relative humidity (R.H.) from 20% to 100%. Similarly, L. Ascherl et al. reported orienting a thin film formed by a tetrakis(4-aminophenyl)pyrene-based COF (Py–TT COF) (Figure 5b) [53]. The thin film exhibited a high humidity sensitivity. UV–Vis spectra revealed that a new absorption band between the 520–640 nm appeared after the exposure to enough H_2_O(g) and the original absorption across the 440–500 nm and 280–380 nm spectral regions simultaneously decreased. The highest sensitivity towards humidity appeared between H_2_O relative pressures of 0.64 and 0.79. Further investigation suggested that the mechanism of the color change is solvatochromism. Notably, the color change from a dry to humid atmosphere cost only 0.21s, and the converse procedure was even faster, when only 0.15 s was needed. It indicated that Py–TT COF thin film was potential material for real-time chromic humidity monitoring. Similarly, S. Jhulki and coworkers also reported a COF (TAPB-−PDA−OH COF) containing 2,5-di(imine)-substituted 1,4-dihydroxybenzene (diiminol) moieties [55]. H_2_O molecules can be adsorbed in the 1D channel of TAPB−PDA−OH COF via hydrogen bond. The adsorption of H_2_O molecules resulted in the color of TAPB−PDA−OH COF changing from orange to dark red (Figure 5c). The response to the humid air cost only 9 s and the recovery procedure needed less than 1 s, indicating that TAPB−PDA−-OH COF is also a good candidate for real-time humidity sensing.

In addition to color change, the adsorption of water vapor can also induce a decrease in impedance. H. Singh and coworkers synthesized truxene-based COFs with boron ester linkages (COF−TXDBA) [56]. The empty orbitals of the boron atom in the COF skeleton allowed the adsorption of H_2_O via the Lewis acid–base interaction with the electron lone pair of the oxygen atom in the H_2_O molecule. When R. H. was low, the amount of H_2_O molecules was too low to form a continuous layer, hindering the charge transportation process among the water molecules by the Grotthuss chain reaction (H_2_O + H_3_O^+^ → H_3_O^+^ + H_2_O). The conductivity of the COF−TXDBA was very low. However, if the amount of H_2_O molecules allowed the formation of the continuous water layer and occurrence of the Grotthuss chain reaction, the impedance would sharply decrease. A linear relationship between R. H. and the logarithm of the impedance can be found in the range of R. H. 11 to 98%. Compared with the above-mentioned chromic method, the humidity sensing based on electrical impedance reported in this study was more quantitative and accurate. However, this approach also displayed a shortage. Its response time was measured as about 40 s. which is much longer than that of the chromic humidity sensor mentioned above.

#### 3.1.3. Harmful Gases Sensing

With the development of the industry, the number of pollution incidences resulting from harmful gases has been increasing in recent decades. For example, the huge coal or oil consumption releases massive amounts of sulfur oxides and nitrogen oxides into the atmosphere, resulting in the high frequency of acid rain. In the indoor environment, the harmful gases released by some shoddy decoration materials such as benzene and formaldehyde are endangering human health seriously. The huge demand for alerting about harmful gases is promoting the development of the COFs for sensing levels of harmful gases.

**Figure 6:**
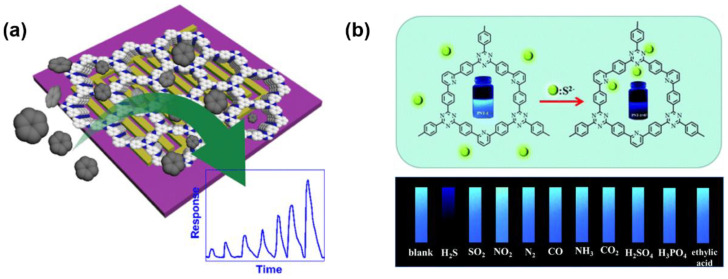
(**a**) COF-based IDE sensor for benzene vapor sensing [58]. Copyright 2020 American Chemical Society; (**b**) COF-based H_2_S detection [71]. Copyright 2017 Royal Society of Chemistry.

As shown in Figure 6a, D. Zhao developed a COF-based chip-like device for benzene vapor sensing [58]. The device was prepared by in situ growing the film of a COF (BTA−TAPT−COF) which was synthesized by the imine condensation between benzene-1,3,5-tricarboxaldehyde (BTA) and 2,4,6-tris(4-aminophenyl)-1,3,5-triazine on an IDE. The exposure to benzene vapor significantly enhanced the capacitance of the device, which can be detected by an LCR bridge. The limit of benzene detection can reach 340 ppb. Notably, due to the interaction between electron-rich benzene and the electron-deficient triazine group, the COF-based benzene sensors showed good selectivity toward benzene over CO_2_, CH_4_, and C_3_H_8_ at room temperature. It suggested that the performance of the capacitive benzene sensor cannot be interfered by common indoor gases.

Among various nitrogen oxides, the detection of NO_2_ drew the most attention, because it is the most likely to appear in the atmosphere. For NO_2_ sensing, the electrical method based on IDE was also applied [64,68,69]. K. A. Mirica and coworkers prepared a COF-coated IDE of which the resistance is sensitive to several harmful gases, including NH_3_, H_2_S, NO, and NO_2_ [64]. The quantitative detection of these gases was realized. The LOD to these gases can reach the level of ppb. However, selective sensing of NO_2_ had not yet been realized. Subsequently, D. Xia and coworkers fabricated a selective NO_2_ sensor by drop-casting a triazine-based 2D organic polymer (T−2DP) on a gold IDE [69]. The T−2DP was prepared by the exfoliation of a covalent triazine framework synthesized through a triflic acid catalytic cyclotrimerization of 1,4-dicyanobenzene. The T−2DP sensor displayed a good performance in sensing trace amounts of NO_2_. The linear range was < 1 ppm and the theoretical LOD is estimated to be 2.2 ppb. The sensor also showed a fast response to the analyte (response time: 35–47 s, recovery time: 56–140 s) and excellent selectivity. This work provided an example of selective high-performance NO_2_ sensing.

In 2017, L. Guo and coworkers reported luminescent organic polymer nanotubes (PNT−1) for S^2−^ sensing [71]. The nanotubes were formed by a 2D structure synthesized by the condensation of 2,4,6-tris-(4-bromo-phenyl)-1,3,5-triazine with 2,6-dibromopyridine. As shown in Figure 6b, the fluorescence of the PNT−1 suspended in CH_3_OH can be quenched by adding Na_2_S. The limit of the “turn off” fluorescent S^2−^ detection of PNT−1 was measured as 53 ppb. Other anions including halide ions, SO_4_^2−^, NO_3_^−^, HSO_3_^−^, PO_4_^3−^, CO_3_^2−^, et al. cannot result in the same phenomenon, indicating the good selectivity of the S^2−^ sensing. However, more importantly, Guo and coworkers subsequently further prepared a test paper for gaseous H_2_S detection by dipping a piece of filter paper into the nanotube suspension for a few minutes (Figure 6b). It was found that the fluorescence of the newly prepared moist test paper can be immediately quenched upon exposure to the H_2_S atmosphere. It indicated that fast detection of gaseous H_2_S can be realized by PNT−1. 

Very recently, a COF-based fast detection of ozone, which is an air pollutant brought by excessive emission of nitrogen oxides, was reported by Z. J. Zhang et al. [72]. The COF used for O_3_ sensing is the frequently-reported TPB−DMTP−COF which was synthesized by 2,5-dimethoxyterephthalaldehyde and 1,3,5-tris-(4-aminophenyl) benzene. In the absence of moisture, ozone can nucleophilically attack imine groups to form nitro, aldehyde, and carboxylate moieties, while in the presence of moisture (R. H. = 40%) imine groups prefer to bind with water molecules to facilitate its protonation, leading to the visible color change from light yellow to red. Furthermore, the author found that TPB−DMTP−COF exhibited a better capacity of ozone removal than other porous materials such as activated carbon, amorphous polymer, monomer, and MOFs.

### 3.2. Inorganic Ions Sensing

#### 3.2.1. Metal Ions Sensing

The concentrations of some metal ions, such as Fe^2+^, Zn^2+^, Mg^2+^, et al., are important indicators of the health of human beings. For example, the analysis of Fe^2+^/Fe^3+^ has great significance in the diagnosis of anemia. Beyond that, heavy metal ions, such as Cu^2+^, Hg^2+^, Pb^2+^, et al., are important industrial pollutants. Excessive heavy metal ions in water sources, food, or drink can cause serious poisoning and irreversible damage to human bodies. Therefore, the simple, rapid, accurate, and sensitive detection of all kinds of metal ions is widely expected in the fields of healthcare, environmental protection, and the food industry. Owing to the unique features of structural designability and selective absorbability, a variety of COFs have been designed for sensing a wide range of metal ions (Table 3).

**Figure 7:**
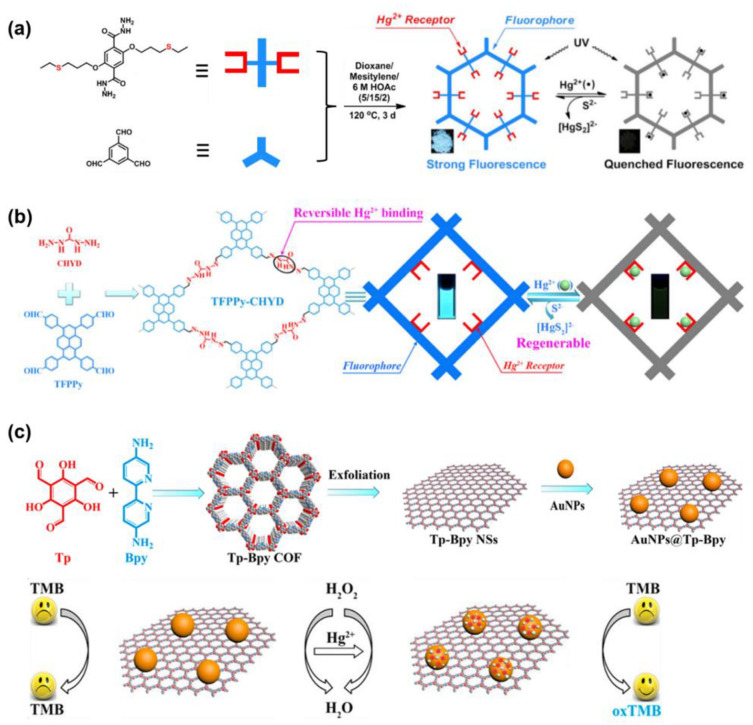
(**a**) COF−LZU8 for Hg^2+^ detection via fluorescence quenching [42]. Copyright 2016 American Chemical Society; (**b**) TFPPy−CHYD for Hg^2+^ detection via fluorescence quenching [73]. Copyright 2020 American Chemical Society; (**c**) Tp−Bpy NSs for Hg^2+^ detection via catalytic oxidation of TMB [29]. Copyright 2019 American Chemical Society.

Hg^2+^ is highly toxic and its existence in natural water or food must be particularly monitored. In 1956, the bioaccumulation of Hg^2+^ from industrial discharge caused the emergence of Minamata disease and finally resulted in a well-known social pollution nuisance. Therefore, many efforts have been devoted to the sensitive detection of Hg^2+^ in water. In general, for designing a COF that can effectively capture Hg^2+^, special groups that can particularly bind with Hg^2+^, such as thiol or thioether, were usually introduced [29,42,73,74]. The high affinity between S and Hg can significantly enhance the capacity of mercury uptake. In 2016, W. Wang et al. reported a fluorescent COF (COF−LZU8) with a hexagonal pore containing thioether group as a selective Hg^2+^ receptor [42]. The electron transformation from COF−LZU8 to Hg^2+^ can result in a markable fluorescence quenching, and the intensity of fluorescence linearly decreased with the concentration of Hg^2+^ (Figure 7a). The LOD was measured as 25.0 ppb. More importantly, the fluorescence quenching exhibited a significant selectivity, except for Hg^2+^, fluorescence quenching caused by a wide range of metal ions is insignificant. Meanwhile, due to the excellent Hg^2+^ uptake capacity, COF−LZU8 can also be easily used for mercury (II) removal. Moreover, upon the simple treatment with concentrated Na_2_S solution, COF−LZU8 can be easily recovered for the next Hg^2+^ detection and removal. 

In addition to the S(II) containing groups, it was reported that the carbohydrazide group can be also applied for capturing Hg^2+^ in the aqueous solution. J.-D. Qiu et al. designed a pyrene-based luminescent COF (TFPPy−CHYD) with a flexible carbohydrazide (CHYD) linkage [73] (Figure 6b). The CHYD group can effectively bond Hg^2+^ in the tetragonal pore of TFPPy−CHYD by coordination interaction. The maximum adsorption capacity for Hg^2+^ can be as high as 758 mg g^−1^. The binding between Hg^2+^ and CHYD also exhibited good reversibility. The Hg^2+^ captured by TFPPy−CHYD can be effectively removed by 10 equiv. of Na_2_S solution. In the meantime, the binding of Hg^2+^ led to a quick, efficient quenching of the fluorescence which cannot be found in presence of other metal ions. The intensity of the fluorescence decreased linearly with [Hg^2+^] when 0.05 μM < [Hg^2+^] < 4 μM. The LOD was measured as 17 nM.

Due to the high toxicity, the threshold level of mercury in drinking water is as low as 10 nM. Therefore, it is still necessary to develop Hg^2+^ sensors with lower LOD. As well as capturing Hg^2+^ into the pore of COFs, other strategies to detect trace amounts of mercury have been also attempted. For example, J.-D. Qiu and coworkers reported a COF-based nanocomposite for Hg^2+^ sensing for which the LOD can be lower than 1 nM [29]. As shown in Figure 6c, they synthesized bipyridine-containing COF nanosheets, Tp−Bpy NSs, with a regular hexagonal pore structure. Due to the nitrogen-containing bipyridine groups, a large number of AuNPs can be doped onto the Tp−BpyTp−Bpy NSs by in situ growth method to form a AuNPs@Tp−Bpy nanocomposite. In citric acid-phosphate buffer solution, AuNPs@Tp−Bpy can adsorb trace amounts of Hg^2+^ from the sample solution to form a gold amalgam layer on the surface of the AuNPs. The amalgam layer allows H_2_O_2_ to be decomposed efficiently to form highly reactive hydroxyl radicals which can rapidly oxidize colorless 3,3′,5,5′-tetramethylbenzidine (TMB) to its bright blue oxidation state (oxTMB). Hence, the absorbance at 652 nm caused by oxTMB can be used to quantitatively determine the concentration of Hg^2+^ in the sample solution. Notably, this approach for Hg^2+^ sensing exhibited an ultrahigh sensitivity. The color changes caused by even 1 nM Hg^2+^ were easily detected.

Apart from Hg^2+^, the detection of Cu^2+^, Fe^3+,^ and Pb^2+^ has also drawn much attention recently. Although the toxicity of Cu^2+^ is far less than Hg^2+^, the widespread existence of Cu^2+^ may still risk the health of citizens. Therefore, COFs that can sensitively and selectively detect Cu^2+^ have been developed. The strategy to develop COFs for Cu^2+^ detection had much in common with COF-based Hg^2+^ sensing. In recent years, several luminescent COFs, of which fluorescence can be quantitatively quenched by the addition of Cu^2+^, were presented. In 2016, X. Liu and co-workers synthesized a hydrogen bond-assisted azine-linked COF (COF−JLU3) with robust chemical stability and thermal stability [75]. It displayed a high luminescence efficiency due to the bulky *tert*-butyl groups on the skeleton, which can adjust the π–π interaction between the layers. The numerous N atoms and hydroxyl groups in the pore of COF−JLU3 acted as receptors to benefit the uptake of metal cations. It was found that transition-metal ions with different electron configurations such as Fe^3+^, Co^2+^, especially Cu^2+^, can be captured resulting in significant quenching the fluorescence of COF−JLU3. The decrease of fluorescence emission intensity was almost proportional to [Cu^2+^] in the range from 0 to 0.4 mM, suggesting that COF−JLU3 is a suitable candidate for Cu^2+^ detection. The LOD of Cu^2+^ sensing was measured as 0.31 mM. In 2018, D. Jiang and coworkers designed and successfully synthesized a series of stable light-emitting two-dimensional sp^2^ carbon-conjugated COFs (sp2c-COFs) [77]. They exhibited excellent luminescence activity and high stability simultaneously. Interestingly, the -CN side groups in sp2c-COFs were able to interact with Cu^2+^ resulting in the quenching of the luminescence. Only 88 ppb (about 1.4 μM) Cu^2+^ can result in an observable quenching. It indicated that sp2c-COFs can serve as a highly selective and effective sensor for detecting Cu^2+^ ions. Later, H. Wang et al. reported a fluorescent QG−scaffolded COF synthesized by the one-step covalent reactions of melamine-aldehyde and phenol-aldehyde poly-condensations using paraformaldehyde, initially with Q-Graphene (QG) scaffolds [78]. The N atoms and hydroxyl groups on the COF skeleton can effectively chelate Cu^2+^. Different from other metal ions, Cu^2+^ can significantly quench the fluorescence of QG−scaffolded COF, indicating that the QG−scaffolded COF is a good candidate for Cu^2+^ sensing. The linear range of the Cu^2+^ sensing was measured as 0.0010~10.0 μM and the LOD can reach 0.50 nM. Moreover, as with the Hg^2+^ detection, this strategy relies on the enzyme-mimicking property of nanomaterials can be also applied in Cu^2+^ detection. In 2017, Y. Xiong et al. developed a covalent triazine framework (CTF) that can exhibit peroxidase-like catalytic activity once it is coordinated with Cu^2+^ [50]. The copper ions acted as the active sites in the complex formed by CTF and Cu^2+^ (CTF/Cu^2+^). It can be found that the catalytic activity of CTF/Cu^2+^ can be tuned by the concentration of Cu^2+^. Upon the addition of H_2_SO_4_, H_2_O_2_, and TMB, the concentration of Cu^2+^ can be easily determined by UV-Vis absorbance. This colorimetric method showed good selectivity. Its linear range was measured as 1.0 μg/L to 80.0 μg/L. and the LOD was determined as 0.05 μg/L.

Iron is one of the essential trace elements of the human body. It plays an important role in oxygen uptake and transfers into red blood cells. Iron is also an important part of some enzymes, for example, catalase. Hence the detection of Fe^3+^ has important significance in the food industry and health examination. In recent years, a number of COFs that can be used for Fe^3+^ detection were reported [79,80,81,82,83,84,85]. Most of them were based on Ion-induced fluorescence quenching. In 2017, W. Yang and coworkers reported two polyimide-based porous COFs (PI−COF 201 and PI−COF 202) [79]. Owing to the p*-n transition caused by the high electro-delocalization and the inherently rigid structure, the two COFs can emit strong fluorescence. However, the adsorption of Fe^3+^ remarkably quenches their fluorescence due to the energy transfer from the emission level of the COFs to the unoccupied d-orbital of Fe^3+^. A linear relationship between the fluorescence intensity and [Fe^3+^] was found in the range of 5.0–40 μM. The LODs were determined as 0.13 μM (PI−COF 201) and 0.22 μM (PI−COF 202), respectively. Later, several luminescent COFs of which fluorescence can be quenched by Fe^3+^ were reported [80,81,82,83,84,85]. The LOD of the COF-based Fe^3+^ sensing has been decreased to 64 nM. Notably, some COFs for Fe^3+^ sensing can be also used to detect other ions, such as Pb^2+^ [85], CrO_4_^2−^, Cr_2_O_7_^2−^, and MnO_4_^−^ [80]. Meanwhile, besides fluorescence quenching, COF-based Fe^3+^ sensing in another manner has been also reported. In 2021, W.-G. Zhang and coworkers prepared a robust hydrazone-linked COF (Tfpa–Mth COF), of which its fluorescence can be quenched by Fe^3+^ [84]. In the meantime, W.-G. Zhang and coworkers prepared a uniform Tfpa–Mth COF thin film on an amino-modified quartz crystal microbalance (QCM) chip by in situ growth method. The Tfpa–Mth COF thin film can capture Fe^3+^, resulting in a frequency shift in QCM measurement. Compared with the fluorescence quenching, the Tfpa–Mth COF-coated QCM chip displayed a significant advantage in real-time Fe^3+^ monitoring. 

Pb^2+^ is another metal ion with which COF-based sensing has been widely concerned. Similar to mercury, lead can be also accumulated in plants and animals and further threaten human beings through the food chain, resulting in irreversible damage to the liver, nervous or circulatory system. Even nowadays, lead is still widely used in batteries, gasoline additives, and paint pigments. Therefore, fast and sensitive lead sensing is still needed. Unlike Hg^2+^ sensing, the reported COF-based Pb^2+^ sensors were more likely to depend on electrochemical signals [85,86,87,88]. In 2018, Y. Wang and J. Wang [86] and co-workers mixed TAPB−DMTTAPB−DMTP−COF [85] with graphite powder to prepare a TAPB−DMTTAPB−DMTP−COF-based carbon paste electrode (TAPB−DMTTAPB−DMTP−COF/CPE) for sensitive and selective Pb^2+^ sensing. The huge surface area and a large number of amino groups of TAPB−DMTTAPB−DMTP−COF greatly benefited the capture of Pb^2+^ from the sample solution. By using TAPB−DMTTAPB−DMTP−COF/CPE, the concentration of Pb^2+^ can be determined by differential pulse stripping voltammetry. The change of current intensity showed excellent linearity to the concentration of lead in the range of 0.0050 to 2.0 μM and the LOD of this method was measured as 1.9 nM. Similarly, X. Wang and coworkers prepared an Au electrode coated with sulfhydryl-modified TAPB−DMTTAPB−DMTP−COF for Pb^2+^ detection [87]. The [Pb^2+^] was determined by square wave anodic stripping voltammetry with the electrode. Benefiting from the affinity between S(II) and Pb, the LOD of the Pb^2+^ detection can be as low as 0.015 ng/mL (0.072 nM). Recently, Y. Zhang et al. further enhanced the sensitivity of COF-based Pb^2+^ detection by introducing the photoelectrochemical (PEC) sensing technique [88]. They firstly prepared a photocathode by fabricating a porphyrin-based COF (TAPP−COF) on a PET-ITO electrode. The photocathode presented an outstanding photovoltaic property owing to the unique charge channels of COFs and the good photoelectric properties of porphyrin. Then the CdSe quantum dots coated with a SiO_2_ shell (CdSe@SiO2QDs), which herein acted as a quenching agent, were immobilized onto the TAPP−COF thin film through a hybridization chain reaction, significantly decreasing the photocurrent. However, the presence of Pb^2+^ can detach the CdSe@SiO2QDs from the TAPP−COF thin film, leading to the quantitative recovery of the photocurrent. Hence, accurate sensing of Pb^2+^ can be achieved in a wide detection range of [Pb^2+^] (0.05~1000 nM) with an extremely low LOD of 0.012 nM. 

**Figure 8:**
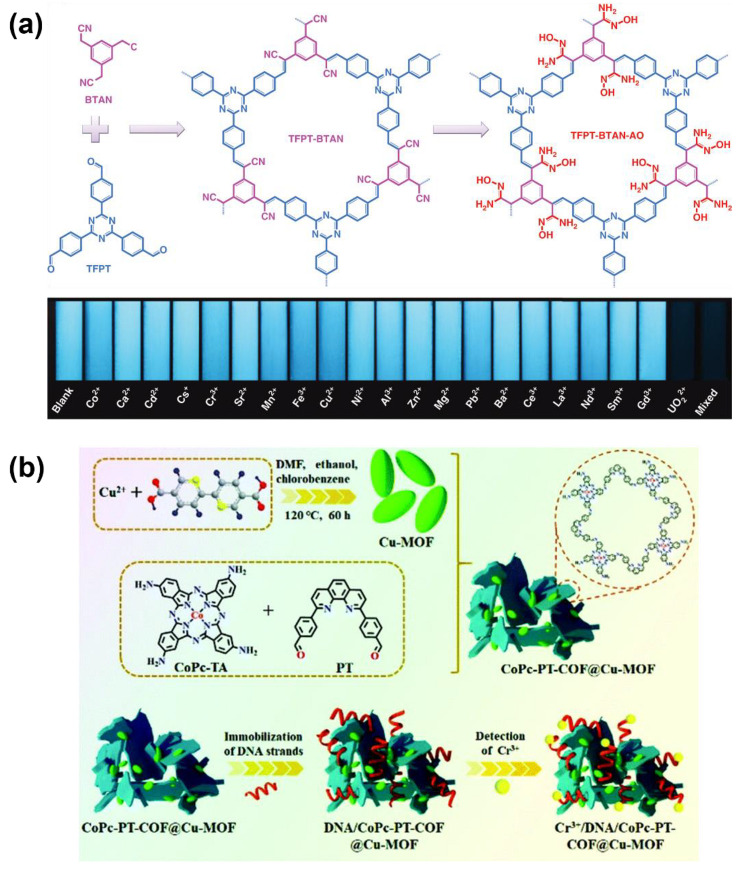
(**a**) TFPT−BTAN−AO for UO_2_^2+^ detection via fluorescence quenching [90]. Copyright 2020 Nature Publishing Group; (**b**) DNA/CoPc−PT−COF@Cu−MOF nanocomposite for Cr^3+^ detection [93]. Copyright 2021 Royal Society of Chemistry.

In recent years, the interest in developing COF-based metal ion sensing has been gradually extended. In addition to the above-mentioned metal ions, the COFs for sensing other ions such as Au^+^, UO_2_^2+^, Ni^2+^, Cr^3+^, Pd^2+^, et al. have been developed one after another. In 2018, Y. Yu designed and synthesized a thioether-functionalized fluorescent COF (TTB−COF) for selective sensing Au ions [89]. Observable fluorescence quenching of TTB−COF can be induced by adding Au ions. The fluorescence intensity and the concentrations of Au ions showed a linear relationship in the range of 1.0–10.0 mM and the LOD is calculated to be 0.87mM. In addition, TTB−COF exhibited a large capture capacity to Au ions (560 mg g^−1^); thus, it is also a promising material for the recovery of ultra-low concentration Au ions in the aqueous solution. Uranium is one of the essential raw materials in the nuclear industry. However, its content in the earth’s crust is quite low, therefore, porous materials for uranium extraction from seawater and detection of uranium have been recently focused on [90,96,97,98,99]. J.-D. Qiu and coworkers prepared a fluorescent COF (TFPT−BTAN−AO) containing a large amount of selective uranium-binding groups as shown in Figure 8a [90,91]. The TFPT−BTAN−AO can selectively capture UO_2_^2+^ ions. The uptake capacity was measured as 427 mg g^−1^, suggesting that it is possible to be applied for the purposes of uranium extraction. The fluorescence emission of TFPT−BTAN−AO linearly decreased upon the gradual addition of UO_2_^2+^. The LOD was determined as 6.7 nM. Similarly, Q. L. Deng and coworkers reported that a COF containing the bipyridine group in its skeleton of fluorescence can be selectively quenched by Ni^2+^ when pH = 10 [92]. As a platform for Ni^2+^ detection, an extremely low LOD of (68 pM) can be achieved. Meanwhile, the COFs have been also used for chromium sensing. As mentioned above, the COF for Fe^3+^ sensing reported by Z. Shi et al. [80] can also be applied for the detection of Cr(VI), which is a highly toxic carcinogen. Meanwhile, M. Du and coworkers prepared a COF-containing nanocomposite as a platform for the high-efficiency determination of Cr(III) [93]. As shown in Figure 8b, the nanocomposite (DNA/CoPc−PT−COF@Cu−MOF) was prepared by firstly growing two-dimensional phthalocyanine-based COF on Cu-MOF and then immobilizing DNA strands that can recognize Cr^3+^. The DNA/CoPc−PT−COF@Cu−MOF was coated onto a glass carbon electrode (GCE), yielding a Cr^3+^ sensor. It was found that the binding with the Cr^3+^ enhanced the charge transfer resistance, R_ct_, of the DNA strands, which can be detected by electrochemical impedance spectroscopy (EIS). In the meantime, DNA/CoPc−PT−COF@Cu−MOF also displayed a photoelectrochemical property. Cr^3+^ can result in a decrease in the photocurrent. Hence the Cr^3+^ can be detected by monitoring both R_ct_ and photocurrent. The calculated LOD of the Cr^3+^ biosensor was as low as 14.5 fM. Very recently, two fluorescent COFs that can selectively detect Pd^2+^ were reported by J.-Y. Yue et al. and Y. Lu. et al., respectively [94,95]. Their fluorescence can be selectively quenched by Pd^2+^. Interestingly, the Pd^2+^-sensitive COF by Y. Lu. et al. was prepared in situ on a piece of paper, yielding a COF-based test paper for Pd^2+^ detection. Considering the wide application of palladium in the industry, it can be expected that the Pd^2+^ test paper will show good practicability in the fast detection of the catalyst residual.

#### 3.2.2. pH Sensing (H^+^ Sensing)

Similar to the detection of the acidic/alkaline gases, introducing an acid/alkaline responsive group is the key to creating a COF for pH sensing (H^+^ sensing in water). [100,101,102,103,104] Early in 2016, Y. Mu and X. M. Liu et al. reported a COF (COF−JLU4) with protonatable β-ketoenamine in its skeleton [104]. The fluorescence spectra of COF−JLU4 can gradually change with the pH value in aqueous solution, indicating that pH-responsive luminescent COFs can be a candidate for pH sensing. Subsequently, several COFs of which fluorescence is responsive to pH value were developed (Table 4) [101,103,104]. Notably, C. L. Zhang et al. synthesized an imine-linked fluorescent COF containing triazine groups (COF_2_) [100]. The protonation of both imine linkage and triazine affected the intensity of fluorescence. Interestingly, the plot of fluorescence titration vs. pH showed a significant jump when pH = 6.5. It is suggested that COF_2_ can be used in the detection of cancer as a fluorescent probe. Thus, C. L. Zhang et al. modified the 2D nanosheet of COF_2_ with poly-D-lysine to improve its dispersity in aqueous solution, biocompatibility, and endocytosis efficiency. The experiment result exhibited that COF_2_ modified by poly-D-lysine can be successfully used in the in vitro cancer cell imaging and in vivo pH imaging in zebrafish.

In addition to fluorescent quenching, COF-based pH sensing can be conducted in other ways. For example, L. Wang and coworkers synthesized an imine-linked COF (COF_DHTA-TTA_) with multiple redox-active states [102]. Owing to the redox activity, the COF_DHTA-TTA_, showed a good performance in catalyzing the decomposition of H_2_O_2_. Therefore, it can be expected that a COF_DHTA-TTA_ coated glassy carbon electrode (GCE) can be applied in the electrochemical analysis of H_2_O_2_. Additionally, the electron transport process of COF_DHTA-TTA_/GCE was accompanied by H^+^ transfer. Thus, pH could be also determined by using COF_DHTA-TTA_/GCE based on the peak potential as signal outputs. The experiment result showed that the peak potential exhibited a good linear relationship with the pH value of the solution in the experiment. In addition, the authors loaded glucose oxidase into the pores of COF_DHTA-TTA_. It made the COF_DHTA-TTA_ coated GCE give a good performance for glucose detection.

#### 3.2.3. Inorganic Anions Sensing 

The inorganic anions in aqueous solution mainly include various acid radical ions. However, the COF for sensing inorganic anions was not frequently investigated as cations. Only a few reports can be found (Table 5). As mentioned above, the Fe^3+^ sensitive COF reported by Z. Shi et al. [80] can also be used for detecting CrO_4_^2−^, Cr_2_O_7_^2−^, and MnO_4_^−^ via fluorescence quenching. The detection of Cr(VI) is of practical value because it is a water pollutant with significant carcinogenicity. So far, the inorganic ion that attracted the most attention is F^−^ [103,104,105], because excessive F^−^ in drinking water can damage human teeth and bones. 

**Figure 9:**
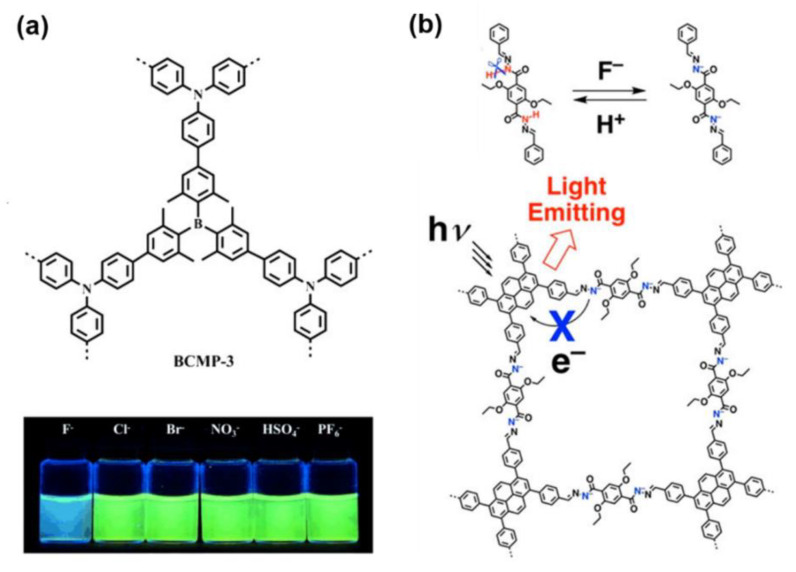
(**a**) BCMP−3 for F^−^ sensing [109]. Copyright 2015 John Wiley and Sons; (**b**) TFPPy−DETHz−COF for F^−^ sensing [110]. Copyright 2018 American Chemical Society.

Early in 2015, X. M. Liu et al. reported on a boron-containing COF (BCMP−3) [105]. Unlike other anions, the Lewis acid–base interaction between the B atom and F^−^ can result in the change of fluorescence of BCMP−3 (Figure 9a). A remarkable blueshift and intensity decrease can be found upon the addition of F^−^. The BCMP−3 can be also used for F^−^ removal. The adsorption capacity was 24 mg/g. Subsequently, D. L. Jiang and his coworkers synthesized a luminescent COF (TFPPy−DETHz−COF) that can be used for F^−^ sensing [106]. They found that F^−^ can react with the N−H unit in the hydrazone linkage of TFPPy−DETHz−COF based on an acid−base reaction mechanism, while other halogen anions like Cl^−^, Br^−^, I^−^ were inert (Figure 8b). The reaction with F^−^ deprotonated the N−H bond to form an anionic nitrogen species which can eliminate the nitrogen-related fluorescence quenching pathway, leading to a remarkable enhancement of the fluorescence intensity. Therefore, TFPPy−DETHz−COF was a rare fluorescence switch-on sensor because its intensity increases as [F^−^] is increased. The detection limit of F^−^ detection was down to 50.5 ppb. Additionally, the F^−^ sensing based on the analogues of COFs, covalent triazine frameworks (CTF), was also reported. The F- sensing can be conducted by the CTF in a pathway without fluorescence. In the same year, Y. H. Xiong demonstrated an iron-modified two-dimensional CTF (2D−Fe−CTF) which exhibited remarkable peroxidase-like activity. F^−^ can coordinate with the iron ion in the CTF and significantly decreased the enzyme-mimicking activity [107]. Therefore, the concentration of F^−^ can be detected by monitoring the rate of H_2_O_2_ decomposition. The LOD of this approach can be as low as 5 nM, and other anions such as Cl^−^, Br^−^, SO_4_^2−^, NO_3_^−^, HCO_3_^−^, HCOO^−^, CO_3_^2−^, PO_4_^3−^, and CH_3_COO^−^ did not show significant interference. 

In addition to F^−^, the COFs for sensing S^2−^ in solution have also been focused on [71,108], because H_2_S can act as a signal transmission molecule in the human body, the same as NO and CO [109,110], and participates in some important pathophysiologic processes such as neuromodulation and vasodilation. As demonstrated above, the organic polymer nanotubes PNT−1 can be used for detecting Na_2_S in CH_3_OH solution. Its fluorescent emission decreased with the addition of Na_2_S. However, for sensing the H_2_S in living bodies, it is still necessary to develop the COF that can respond to the trace amount of H_2_S (HS^−^, S^2−^) in aqueous solution. In 2018, P. Wang et al. reported a COF-based hybrid probe, TpASH-NPHS, for targeting H_2_S around the living cells [108]. The hybrid probe was prepared by covalently anchoring a two-photon fluorescent probe 4-amino-1,8-naphthalimide derivative (NPHS) onto an imine-linked COF, TpASH. To further enhance the dispersibility, the micron-sized, bulky TpASH-NPHS was treated by solvent-assisted liquid sonication. The resulting nano-sized TpASH-NPHS can be well dispersed into the aqueous solution. The azide units on NPHS can be reduced to NH_2_ in the presence of H_2_S (HS^−^, S^2−^), which will increase the fluorescence emission of NPHS. Thus, TpASH-NPHS showed a good performance in two-photon fluorescence imaging of H_2_S (HS^−^, S^2−^) in live tumor cells and deep tumor tissues. The hybrid fluorescent probe TpASH–NPHS was able to detect the concentration of H_2_S (HS^−^, S^2−^) in the range of 0–20 mM with a LOD of 0.11 mM. Furthermore, TpASH–NPHS was inert to a wide range of interferents, including common anions, biothiols, cysteine, homocysteine, and reactive oxygen species. Notably, since the NPHS was immobilized in the 1D channel of which the diameter is about 1.5 nm, the H_2_S (HS^−^, S^2−^) imaging by TpASH–NPHS was not interfered by some intracellular enzymes (diameter > 3 nm), which are also able to reduce the azide group under hypoxic conditions. Hence, this COF-based hybrid probe displayed a great promise in clinical applications.

### 3.3. Molecular Sensing

#### 3.3.1. Explosive Sensing

Explosive detection is widely required in the security of airports, railway stations, and border checks. Therefore, plenty of effort has been devoted to the fast, portable detection of explosives. Various COFs that can be used for explosive sensing have been developed (Table 6) [76,111,112,113,114,115,116,117,118,119,120,121,122,123,124,125,126,127,128,129,130].

Nitroaromatics, the most widely used explosive, have an electron-deficient aromatic ring which is an effective fluorescence quencher. It can be expected that the fluorescence of some COFs can be easily quenched by adsorbed nitroaromatics. Therefore, the key point to realizing COF-based nitroaromatics detection is designing a fluorescent COF that can effectively adsorb nitroaromatics. Early in 2012, L.-G. Qiu and coworkers demonstrated a melamine-based COF (SNW−1) for trace-level detection of nitroaromatic explosives [111]. The melamine-based fluorescent COF was synthesized by the microwave-assisted reaction between terephthalaldehyde and melamine. It was found that the fluorescent emission of SNW−1 can be quenched after adsorbing nitroaromatics due to the transfer of photoexcited electrons from the excited luminescence network donor to the electron-deficient nitroaromatic acceptors. In the THF–water (1:9, *v*/*v*) mixture, the intensity of the fluorescence linearly decreased with the concentration of nitroaromatics including nitrobenzene, 4-nitrotoluene, 4-nitrophenol, 2,4-dinitrotoluene (DNT), picric acid, 2,4,6-trinitrotoluene (TNT), and 2,4,6-trinitrophenylmethylnitramine. The LODs for TNT and DNT were 1.51 × 10^−6^ mol L^−1^ and 2.8 × 10^−6^ mol L^−1^, respectively. Remarkably, the LOD for picric acid can be as low as 5.0 × 10^−8^ mol L^−1^ (11.5 ppb). Furthermore, L.-G. Qiu and coworkers also applied SNW−1 to detect the vapor of DNT, which is a volatile by-product of commercial TNT. It can be found that the fluorescence of SNW−1 decreased by 50% within 10 s, suggesting an ultrafast response to DNT. The LOD of SNW−1 for DNT vapor detection was calculated to be 9.8 ppb, much lower than most DNT detectors that had been reported.

**Figure 10:**
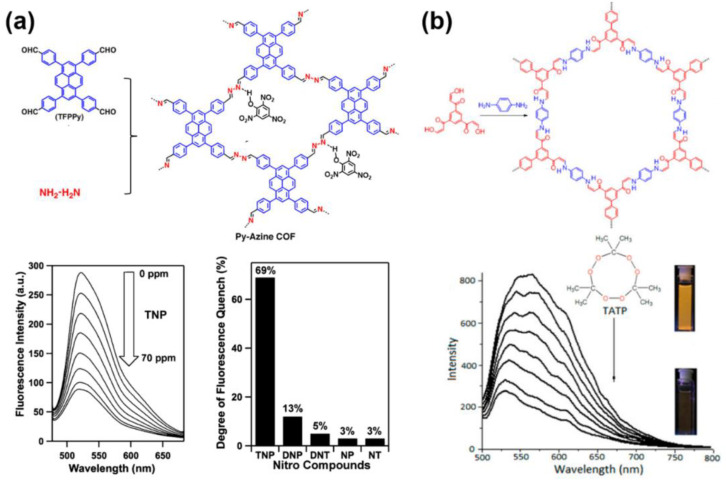
(**a**) The azine-linked COF for picric acid (2,4,6-trinitrophenol) detection. 2,4,6-trinitrophenol (TNP), 2,4-dinitrophenol (DNP), 2,4-dinitrotoluene (DNT), 2-nitrophenol (NP), and 2-nitrotoluene (NT) [116]. Copyright 2013 American Chemical Society; (**b**) The COF for TATP sensing [115]. Copyright 2017 American Chemical Society.

In the following years, many COFs of which the fluorescence can be quenched by nitroaromatics were found. For example, D. Jiang and coworkers reported an azine-linked COF, of which the fluorescence can be quenched by some nitroaromatics like picric acid, 2,4-dinitrophenol (DNP), DNT, 2-nitrophenol, and 2-nitrotoluene (Figure 10a) [109]. It can be also found that the quenching result from picric acid was much more significant than other nitroaromatics. The authors demonstrated the strong quenching effect can be attributed to the hydrogen bond formed by the -OH group of the analytes and the open N atoms on the COF skeleton. In addition, the three nitro groups of picric acid resulted in the most deficient π system for driving the fluorescence quenching and the strongest hydrogen bond with the azine for promoting the quenching process. After the reveal of the mechanism, many COFs for picric acid detections were successfully designed and synthesized [112,113,114,115,116,117,118,119,120,121,122,123,124,125,126,129,130]. The LOD has been decreased to ~10 nM (Table 6). Furthermore, the interference from other nitroaromatics especially dinitrophenol and nitrophenol has been reduced to the largest extent [112,113,114]. Moreover, considering that other nitroaromatics including dinitrophenol and nitrophenol are also explosive, it can still be concluded that the fluorescence quenching strategy based on COFs is an effective method for the nitroaromatic explosive initial inspection.

In addition, the COF-based detection of non-nitroaromatic explosives has been also presented. In 2018, D. F. Perepichka synthesized two kinds of fluorescent COFs (3BD and 3′PD) via Michael Addition−Elimination [115]. The fluorescence of the two COFs cannot only be quenched by nitroaromatics like picric acid but is also sensitive to triacetone triperoxide (TATP), which is another well-known explosive. The quenching caused by TATP was possibly attributed to the oxidation of the enamine moieties in 3BD and 3′PD (Figure 10b). A total of 1 μM TATP solved in CH_2_Cl_2_ can be detected. The authors suggested that the quench should not result from the oxidation of the enamine moiety in the COF skeleton rather than the electron transfer from excited COFs to the analyte, like nitroaromatics.

#### 3.3.2. Iodine Sensing

Radioiodine (^129^I and ^131^I), which is generated by nuclear fission, is an important index for detecting nuclear fallout. It is pernicious to the human body because of its long radioactive half-life (^129^I, 1.57 × 10^7^ years) [131] and high volatility. Therefore, it is widely concerned to detect radioiodine fast and sensitively. COF and its analogs have been proved to be excellent adsorbents for iodine removal. For example, TPB−DMTP−COF synthesized by D. L. Jiang and coworkers can effectively adsorb I_2_ vapor with an adsorption capacity of 6.2 g/g [132]. As with nitroaromatics, the molecule of iodine is an electron-deficient species. The charge transfer from the electron-rich COF skeleton to the iodine can significantly quench the fluorescence of the COF. Thus, it is very likely to achieve “turn off” fluorescence sensing to iodine by COF or its analogs. Recently, a number of iodine sensors based on COPs was presented [133,134,135,136,137,138,139]. In 2020, T. M. Geng et al. prepared a fluorescent covalent triazine-based framework (TDPDB) by Friedel–Crafts polymerization reaction of N, N−-diphenyl-N, N−-di(m-tolyl)benzidine (DPDB) with 2,4,6-trichloro-1,3,5-triazine (TCT) [140]. It exhibited an excellent capacity for capturing iodine (3.93 g/g). In the meantime, the adsorption of iodine quenched the fluorescence of TDPDB remarkably. The quenching coefficient (Ksv) was measured as 5.83 × 10^4^ L mol^−1^ and the LOD reached 2.57 pM. It indicated that TDPDB can be used for highly sensitive I_2_ detection. Furthermore, the concentration of PA can be also measured by TDPDB based on the same mechanism. The Ksv and LOD for PA detection were determined as 1.55 × 10^4^ L mol^−1^ and 19.3 pM, respectively. The next year, Y. X. Zhao et al. reported a COF (COF-PA) with acetylene groups in its 1D channels [131]. The acetylene groups greatly accelerated the adsorption rate of I_2_ vapor. The charge transfer between COF-PA and I_2_ effectively decreased the intensity of the fluorescence of COF-PA. I_2_ at ppm level in THF solution can be well detected by the fluorescence quenching of COF-PA.

#### 3.3.3. Drug Sensing

Drug sensing has a strong practical value and significance because it is a key essential step in investigating pharmacokinetics, which is quite instructive in clinical treatment. Moreover, the drug residue, especially the antibiotics’ residue, in food and drinkable water because of the abuse of drugs was frequently reported in recent decades. It is likely to result in the appearance of the “superbug” by which the infection caused is incurable because it is resistant to the existing antibiotics. Hence, the fast detection of drugs, especially antibiotics, in bodily fluids, food, drink, and environment attracted increasing attention. COF-based detections of drugs, especially various antibiotics [49,107,140,141,142,143,144,145,146,147,148,149,150,151,152,153] were frequently reported in recent years, especially in the past five years. Notably, as shown in Table 7, it can be found that, unlike gases or ions, most of the COF-based drug detection is accomplished by a hybrid material containing COFs, rather than by a separate COF, because most of the binding sites for the selective capture of the drug molecules, including aptamers, metal ions and so on, were difficult to be robustly pre-immobilized onto the monomers. They are easy to detach from the monomers or be damaged during the solvothermal synthesis of COFs. Hence, they can only be introduced by the “post-functionalization” of the COFs. In addition, in some COF-based sensors for drug detection, the selective capture of drug molecules was not always achieved by the COFs, but by other materials like molecular imprinting membranes.

**Figure 11:**
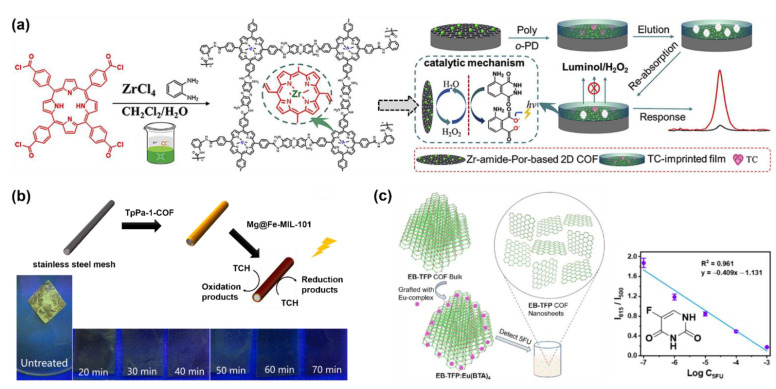
(**a**) Zr-coordinated amide porphyrin-based COF for tetracycline detection with the aid of molecular imprinting technology [144]. Copyright 2019 Elsevier B.V.; (**b**) The preparation of Mg@Fe−MIL−101/TpPa−1−COF, and its fluorescent detection of tetracycline realized simultaneously with photocatalytic tetracycline removal [152]. Copyright 2021 Elsevier B.V.; (**c**) EB-TFP:Eu(BTA)_4_ for the detection of 5-Fluorouracil [154]. Copyright 2021 American Chemical Society.

Tetracycline, a broad-spectrum antibiotic, is widely used in the treatment of bacterial infections in humans and animals. Therefore, tetracycline residues were frequently found in the products of the breeding industry or aquaculture. Thus, as shown in Figure 11a, X. H. Ma synthesized a Zr-coordinated amide porphyrin-based COF by a liquid-liquid interface method [144]. The COF exhibited strong catalytic effects and high electrical conductivity. Hence it was immobilized onto a GCE via the polymerization of the *o*-phenylenediamine to yield an electrochemiluminescence (ECL) sensor. In the meantime, molecular imprinting technology was used to enhance the selectivity of tetracycline. It was found that the intensity of the ECL signal obtained using a luminol-H_2_O_2_ system linearly increased with the concentration of tetracycline. The linear range was 5–60 pM and the LOD was calculated as 2.3 pM. In 2021, Y. S. Zhang reported a COF-containing porous hybrid, Mg@Fe−MIL−101/TpPa−1−COF, grown on stainless steel mesh (Figure 11b) [152]. The porous property of Mg@Fe-MIL-101 exposed more catalytical active sites, and the hybrid structure facilitated the electrons’ migration. Therefore, the Mg@Fe−MIL−101/TpPa−1−COF exhibited excellent photocatalytic tetracycline degradation capacity. Meanwhile, the Mg^2+^ in the hybrid can coordinate with tetracycline and form the fluorescent Mg-tetracycline complex. Hence, fluorescent detection can be realized simultaneously with photocatalytic tetracycline removal (Figure 11b).

Due to the side effects and increasing resistance, the use of antibiotics in the clinic treatment is being updated, therefore, the COF-based antibiotic detection needs to be constantly extended. B. Yan and coworkers prepared a Eu^3+^ functionalized COF hybrid material (Eu@TpPa−1) by simply soaking TpPa-1 COF [158] in a EuCl_3_ solution [145]. It was found that due to the coordination between Eu^3+^ and levofloxacin, Eu@TpPa−1 can acted as a fluorescence sensor toward levofloxacin with a turn-on response. The linear range of the levofloxacin sensing was 1−10^4^ μM, and the LOD was calculated as 0.2 μM. The sensor also displayed a fast response to levofloxacin which was less than 1 min. In the meantime, it also exhibited a good selectivity refraining from the interference of other components in serum and urine. In 2021, S. Wang reported a polyimide COF of which fluorescence can be quenched by levofloxacin, tetracycline, and some metal ions [153]. It exhibited a potential application in the fast identification of multiple harmful substances in drinkable water.

In addition to antibiotics, COF-based sensing of other drugs was also reported (Table 7). For example, 5-fluorouracil (5FU), an anticancer drug, can be detected by a COF hybrid material (EB-TFP:Eu(BTA)_4_) prepared by B. Yan et al. (Figure 11c) [154]. The hybrid material was prepared by soaking a previously reported COF [159] in an Eu complex. The adsorption of 5FU can disturb the π−π stacking interaction between the 2D sheets of EB-TFP:Eu(BTA)_4_), leading to the change in the fluorescent spectra. It resulted in an increase in the fluorescent peak at 615 nm and an decrease in the peak at 500 nm. The intensity ratio I_615_/I_500_ linearly decreased with the logarithm of the concentration of 5FU. Therefore, a 5FU sensing based on fluorescence can be achieved. The linear detection range of the 5FU sensing was 10^−7^−10^−3^ M. The calculated LOD was 5.13 × 10^−8^ M. In addition, B. Yan et al. demonstrated a dye-functionalized COF for ultrasensitive monitoring of sialic acid, which is a widely-used cosmetic additive and biomarker of ovarian cancer. Similar to the above-mentioned Eu@TpPa−1, B. Yan et al. loaded fluorescein sodium (TpPa−1@Dye) into the porous TpPa-1 COF [154]. The TpPa−1@Dye was mixed in sodium alginate solution and finally formed a hydrogel with the assistance of Cr^3+^. The interaction between TpPa−1@Dye and Cr^3+^ caused fluorescence quenching of fluorescein, while the sialic acid can take away Cr^3+^ from the TpPa−1@Dye leading to the recovery of the fluorescence. The fluorescence recovery is fast and very sensitive to the addition of sialic acid, the LOD of the sialic acid detection in serum by this approach can be as low as 8.71 × 10^−9^ M.

#### 3.3.4. Small Biomolecules Sensing

The level of some small biomolecules, including glucose, uric acid, Glutathione (GSH), et al., are key marks of some serious diseases. Hence the detection of them is very important in the healthcare and clinic diagnosis. Recently, with the demands for portable and wearable healthcare devices, the reports on new materials for facile detection of the above small biomolecules have been increasing. Recently, some COFs or COF-based hybrid materials were designed and synthesized for detecting glucose, uric acids, GSH, and other small biomolecules (Table 8) [26,27,102,160,161,162,163].

The concentration of glucose in blood is an essential index for the diagnosis of diabetes. Plenty of methods to detect the concentration of glucose have been developed. The basic strategy to sense glucose is by detecting H_2_O_2_ yielded by the enzymic oxidation of glucose. In 2018, J. N. Wang et al. and co-workers prepared a Fe-porphyrin-based peroxidase-mimicking COF (Fe−COF) [160]. Like the COF demonstrated by J. D. Qiu and coworkers [148], Fe−COF can be used to quantitatively detect the [H_2_O_2_] by the generation of the oxTMP with blue color. Furthermore, upon the mixing of glucose oxidase (GOx), the combination of the enzymic glucose oxidation and Fe−COF-catalyzed oxTMP production resulted in the realization of a glucose sensor. the detection range of the sensor was from 5 to 350 μM. The detection limit was 1.0 μM. On the basis of this principle, several COF-based glucose sensors with improved properties were developed [102,161,162]. For example, the function of the pH sensor mentioned above by L. Wang and coworker can be extended for glucose sensing [102]. Recently, J.-Yu Yue et al. prepared a glucose sensor by coupling the GOx on a carboxyl-containing COF via a covalent amide bond [162]. The sensor showed good recoverability and storage stability. Even after 100 days of storage, the relative activity of GOx was still more than 85%. In the meantime, this sensor displayed a wider linear range from 0.005 to 2 mM and a lower LOD which was calculated as 0.54 mM.

The level of uric acid in serum is the most important index for the diagnosis of gout, which is a common ailment in developed countries. In 2021, Y. S. He et al. developed a COF-based electrochemical sensor for detecting uric acid [163]. For fabricating the sensor, a poly-fuchsin basic film was fabricated on the surface of a bare glass GCE. Then Au nanoparticles were doped on a carboxylated COF (ACOF-TaTp), yielding a hybrid, AuNPs@ACOF-TaTp. Finally, the sensor was achieved by dropping the hybrid on the poly-fuchsin basic coated GCE. The COF in the sensor provided a high surface area and numerous binding sites (H-bond binding) for the analyte. The existence of AuNPs enhanced the conductivity, while the poly-fuchsin basic film can accelerate the electron transfer process. Therefore, the sensor exhibited high affinity to the analyte and high sensitivity and selectivity in uric acid sensing. It was reported that there were two linear response ranges of the AuNPs@ACOF-TaTp-based uric acid sensor, 5.0–25 μM, and 25–250 μM. The LOD was calculated as 0.77 μM, (normal uric acid level of human: 89–416 μM) indicating the sensor has the potential to be used in the detection of uric acid in serum. In addition, the sensor can be applied to detect the concentrations of ascorbic acid and dopamine, which are also important biomolecules.

**Figure 12:**
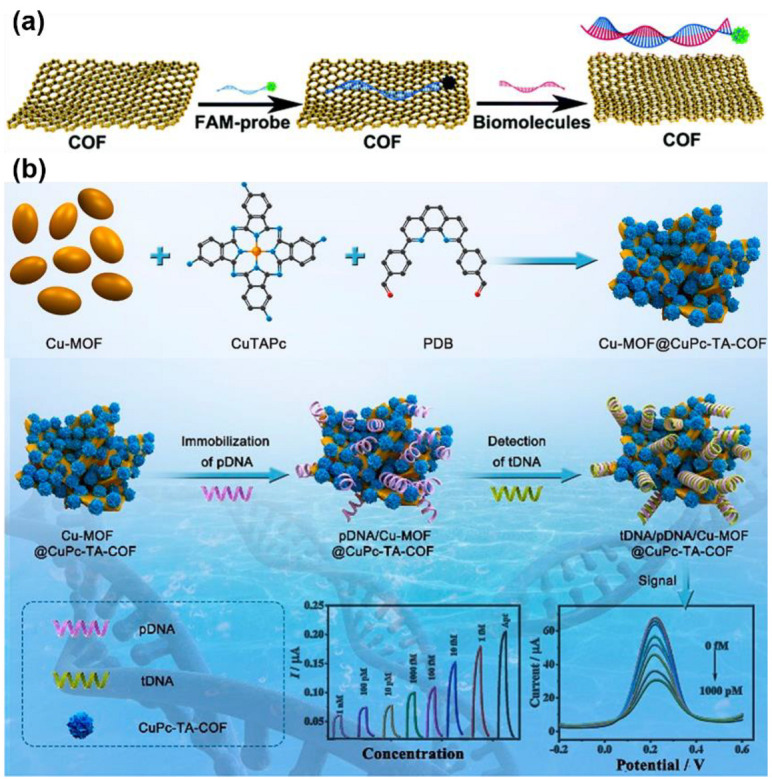
(**a**) COF−300−AR for GSH detection [26]. Copyright 2020 American Chemical Society; (**b**) Py−TT COF for GSH sensing [27]. Copyright 2021 American Chemical Society.

GSH is a biomolecule that widely exists in all kinds of cells in the human body. The excessive glutathione in serum usually suggests hepatocellular injury or tumor. In 2020, H. L. Chen and coworkers reported an amine-linked COF (COF−300−AR) obtained by reducing the corresponding imine-linked COF (COF-300) (Figure 12a) [26]. It exhibited excellent oxidase-mimicking activity under light irradiation of which *λ* = 400 nm, catalyzing the oxidation of TMB by the formation of reactive oxygen species like •OH and O_2_^•−^ free radicals in the presence of dissolved oxygen. However, the reducibility of GSH can prevent the oxidation of TMB and lighten the blue color resulting from oxTMB. Therefore, the concentration of glutathione can be measured by colorimetric analysis. The authors successfully applied COF−300−AR to detect GSH levels in HL60 cells. It displayed good selectivity and high sensitivity. Similarly, Z. Lin et al. synthesized a COF (Py−TT COF) using the condensation between tetrakis(4-aminophenyl)pyrene (Py), which can act as an electron donor, and thieno[3,2-*b*]thiophene-2,5-dicarbaldehyde (TT), which can act as an electron acceptor (Figure 12b) [27]. Like COF−300−AR, Py−TT COF exhibited superior enzymatic catalytic activity, which could catalyze the oxidation of TMB by the formation of ROS under visible light irradiation. Therefore, based on the similar mechanism, the concentration of GSH can be determined by the decrease of the blue color generated by oxTMB. The linear range of this approach was 0.4 − 60 μM, and the LOD was 0.225 μM, satisfying the detection of the GSH in human serum.

#### 3.3.5. Other Small Molecules Sensing

Besides the residue of antibiotics, the detection of insecticides residual is also an important problem in agriculture [155,156]. For insecticide sensing, Y. H. Song et al. synthesized a COF by the imine condensation (DAAQ−TFP) between 2,6-diaminoanthraquinone (DAAQ) and 1,3,5-triformylphloroglucinol (TFP) [155]. DAAQ−TFP exhibited a very good capacity for adsorbing benzoylurea insecticides due to the hydrogen bond and π-π interaction between the benzoylurea and COF skeleton. The benzoylurea insecticides adsorbed by DAAQ−TFP can be eluted by organic solvent for quantitative detection by HPLC. This approach can be applied for detecting insecticide residuals in fruit samples and provided very low detection limits (~0.1 ng mL^−1^).

The COF-based detection of spice, of which concentration is quite important in the food industry, was also investigated. H. L. Liu can co-workers reported a fluorescent nanoprobe (CN-grafted COF@MIPs) for the detection of 4-ethylguaiacol, which is a crucial index to evaluate the quality of some kinds of wine [157]. The probe was a 4-ethylguaiacol imprinted polymer doped with a COF grafted onto carbon nanodots. The molecular imprinting and -NH_2_ in the hybrid material enhanced the selective uptake of the analyte. The adsorption of 4-ethylguaiacol quenched the fluorescence of carbon nanodots through charge transfer interaction. Under optimized conditions, the fluorescence of CN-grafted COF@MIPs drops linearly as the concentrations of 4-ethylguaiacol increase from 0.025 to 1 μg mL^−1^, with a LOD of 17 ng mL^−1^.

Finally, it is worth mentioning that Y. Cui and co-workers reported a chiral 1,1′-bi-2-naphthol-based (BINOL-based) COF for sensing chiral organic compounds [164]. The COF was synthesized by imine condensations of tetrakis(4-aminophenyl)ethene (TPE-TAM) and the chiral linker (R or S) 6,6′-dichloro-2,2′-diethoxy-1,1′-binaphthyl-4,4′-dialdehyde (BINOL-DA). The chiral linker created a 1D channel with high enantioselectivity for the discriminating uptake of chiral analytes. For example, the nanosheets of the COF synthesized by (R)-BINOL-DA can adsorb more (−)-α-pinene than (+)-α-pinene. Thus, the (−)-α-pinene can result in a faster and greater fluorescence quench than (+)-α-pinene at the same concentration. The authors found that the BINOL-based COF nanosheets exhibited much better selectivity and enantio-sensitivity to other BINOL-based sensing systems, probably due to the steric confinement of the COF channels and conformational rigidity of the immobilized BINOL groups.

#### 3.3.6. Biomacromolecule Sensing

Biomacromolecules, including polysaccharides, proteins, nucleic acids, et al., are essential components of living organisms and play an important role in various life activities. The detection of biomacromolecules, especially proteins and nucleic acids, is extensively needed in clinical treatment, public health, and environmental protection. Recently, a number of COFs or COF-containing hybrid materials have been developed for sensing proteins and nucleic acids (Table 9).

Although protein is a kind of biomacromolecule with huge diversity, it is not difficult to specifically enrich the target protein from the sample solution, because most proteins can be recognized and captured by the corresponding antibody. Due to the large specific surface area, COFs are good carriers for immobilizing the antibodies. Moreover, other functional species can be simultaneously introduced onto the COF sheets to produce a detectable signal after the target protein was bound via antigen-antibody recognition. Based on this strategy, some COF-containing sensors for detecting protein with diagnostic significance were developed recently [165,166,167,168,169].

**Figure 13:**
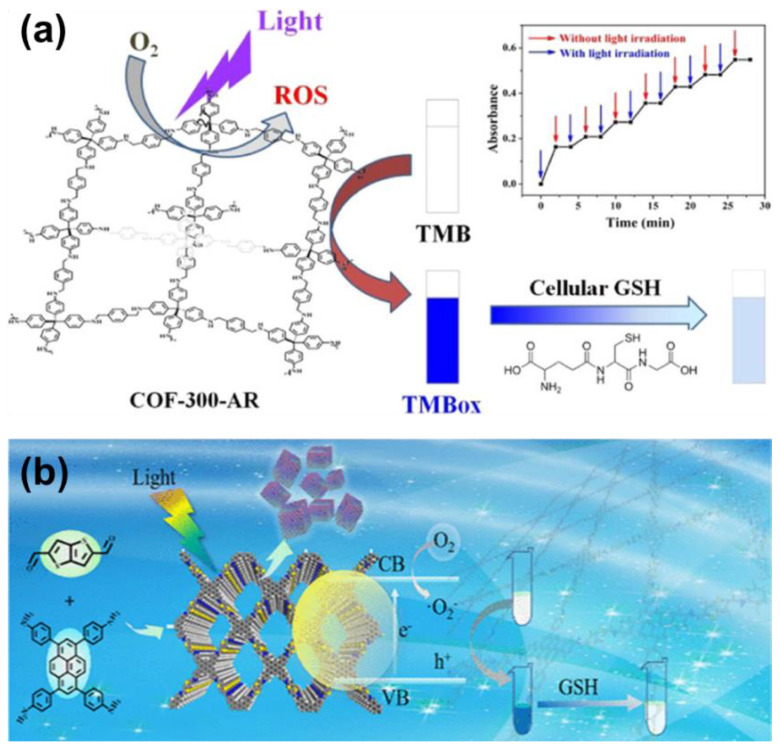
(**a**) Immunosensor for cTnI Detection based on TB−Au−COFs−Ab_2_ [165]. Copyright 2018 Elsevier B.V; (**b**) Immunosensor for cTnI Detection based on HRP−Ab2−Au−COF [166]. Copyright 2021 American Chemical Society.

In 2018, T. Zhang et al. reported a COF-based immunosensor for detecting cardiac troponin I (cTnI), which is an important marker for angina pectoris and myocardial infarction (Figure 13a) [165]. Firstly, AuNPs were loaded into the pore of a previously-reported COF [170], then cTnI secondary antibody (Ab_2_-cTnI) was immobilized onto the AuNPs. Finally, toluidine blue (TB) was loaded into the COF, yielding a COF-based label, TB−Au−COFs−Ab_2_. Meanwhile, a GCE was coated by TiO_2_ NPs which is successively modified by polypyrrole, AuNPs, and cTnI primary antibody (Ab_1_-cTnI). By the specific recognition antigen-antibody, the TB−Au−COFs−Ab_2_, analyte, and modified GCE can form a sandwich-like structure, resulting in a redox signal that can be detected by square-wave voltammetry. The concentration of CTnI can be determined by the intensity of the square-wave voltammetric signal. The authors found that the AuNP-doped COF can amplify the electrochemical signal and enhance the performance of the immunosensor. The immunosensor showed a linear range from 0.5 pg mL^−1^ to 10.0 ng mL^−1^ and a low detection limit of 0.17 pg mL^−1^. In 2021, S. N. Feng et al. reported a similar approach to detect cTnI [166] (Figure 13b). TB in the above work was replaced by horseradish peroxidase (HRP) to fabricate the COF-based label. Meanwhile, Ab_1_-cTnI was modified directly on a gold electrode instead of GCE, avoiding the complex modification procedure. After the formation of the antibody-antigen-antibody sandwich structure, H_2_O_2_ and hydroquinone was added, and the intensity of the redox signal of hydroquinone can be detected by cyclic voltammetry and used to determine the concentration of cTnI.

X. Fang and coworkers prepared a COF-coated Fe_3_O_4_ NPs as an immunoaffinity probe for the detection of heat shock protein 90α (Hsp90α), a biomarker for liver cancer [167]. The probe was prepared by in situ growth of COFs on an -NH_2_ coated Fe_3_O_4_ NPs, and successively modified by Hsp90α antibody and PEG. The probe can selectively capture Hsp90α in the sample and be collected by the magic field. The Hsp90α on the probe was digested with free trypsin and the resulting peptides’ mixture can be collected for further MALDI-TOF-MS analysis to determine the existence of Hsp90α.

Nucleic acid is an essential biomacromolecule in living bodies and the carrier of genetic information. Nucleic acid detection is widely applied in the identification of bacteria and viruses. During the pandemic caused by COVID-19, nucleic acid detection played a huge part in tracking the virus and preventing the spread of the disease. The key feature of nucleic acid is the base pairing. It allows the detection of a particular nucleic acid segment by using its complementary sequence. Based on this principle, some COF-based detection of DNA was developed [28,171,172,173].

**Figure 14:**
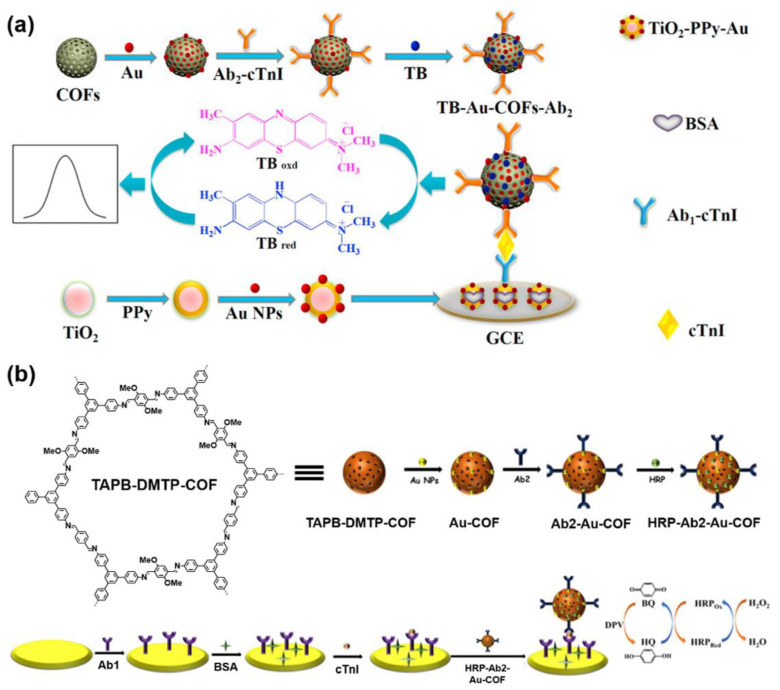
(**a**) A versatile COF-based platform for sensing DNA [171]. Copyright 2017 Royal Society of Chemistry; (**b**) MOF@ COF Heterostructure Hybrid for Dual-Mode Photoelectrochemical−Electrochemical HIV-1 DNA Sensing [173]. Copyright 2021 American Chemical Society.

In 2017, X.-P. Yan demonstrated a versatile COF-based platform for sensing DNA. The sensor is on the basis of the recognition of the complementary DNA sequences and fluorescent quenching (Figure 14a). For detecting a particular DNA sequence (target DNA), its complementary sequence modified with a carboxyfluorescein (FAM) label was adsorbed onto a COF (TpTta) [171] containing triazine, amino- and carbonyl-group via hydrogen bonding, and π-π interaction. The adsorption of the FAM-labeled complementary sequence led to the quench of the fluorescence. However, the hybridization between the target DNA and the FAM-labeled complementary sequence led to the formation of double-stranded DNA that can detach from TpTta, resulting in the fluorescent recovery of the carboxyfluorescein. Moreover, the author found that quantitively ATP detection can be also achieved by replacing the above FAM-labeled complementary sequence with the FAM-labeled adenosine 5′-triphosphate (ATP) aptamer probe. The enhanced fluorescence intensity showed a good linear relationship with the ATP concentration in the concentration range of 25–200 μM and good selectivity.

M. R. Xu et al. developed a novel MOF@ COF hybrid with excellent electrochemical activity and high photoactivity (Figure 14b) [28]. By immobilizing the complementary sequence, the MOF@ COF hybrid can be applied for HIV-1 DNA detection, because the complementary hybridization enhanced the impedance of the hybrid and decreased both the photoelectrochemical and electrochemical currents. Hence, it is possible to achieve a dual-mode detection of HIV-1 DNA in human serum. It was found that both the currents decreased linearly with the increasing lg [HIV-1 DNA], in the range from 1 fM to 1 nM, and the LODs were calculated as 0.07 and 0.18 fM for PEC and DPV techniques, respectively, indicating that the ultra-sensitive sensing of HIV-1 DNA was realized.

**Table 9:** COF-based sensing of some typical biomacromolecules.

Analyte	Year	COF/COF Hybrid Names	Specific Binding Site	Type of Detectable Signal	Detection Range	LOD	Reference
Cardiac Troponin I	2018	TB−Au−COFs−Ab_2_	Antibody	Chromism	0.5 pg/mL–10.0 ng/mL	0.17 pg/mL	[165]
	2021	HRP−Ab2−Au−COF	Antibody	Chromism	5 pg/mL–10 ng/mL	1.7 pg/mL	[166]
Heat shock protein 90α	2019	Fe3O4@TpBD−DSS−Ab−MEG	Antibody	MS		50 pg/mL	[167]
C-reactive protein	2018	AuNPs @COF−TPPa-1	Antibody	Electrochemical signal (EIS, CV)	0.017 ng/mL	0.05–80 ng/mL	[168]
	2018	COF−LZU8	Antibody	Electrochemical signal (DPV)	0.016 ng/mL	0.05–150 ng/mL	[169]
	2019	p−COF	Aptamer	Photoelectrochemical signal	0.1 ng/mL	0.5–100 ng/mL	[174]
DNA	2017	TpTta	DNA hybridization	Fluorescence, “turn on”	10–100 nM	3.7 nM	[171]
	2017	TPA−COF	DNA hybridization	Fluorescence, “turn on”	0.02–5 nM	20 pM	[172]
	2018	EB-TFP iCOF	DNA hybridization	Fluorescence, “turn on”	0–32 mM	-	[173]
	2021	Cu−MOF@CuPc−TA−COF	DNA hybridization	Electrochemical signal	1 fM–1 nM	0.18 fM	[28]
				Photoelectrochemical signal		0.07 fM	

## 4. Challenges and Future Perspectives

In this review, we discussed the basic principles of COF-based chemical and biological sensing, then demonstrated the application of COFs in the detection of a large number of analytes, including corrosive and harmful gases, water vapor (humidity), ions, explosives, drugs, biomolecules, et al. It can be found that COFs exhibited many advantages in chemical and biological sensing. Firstly, due to the designable structure and easily modified properties, the molecular structure and function of the COFs can be easily extended. There is always a good possibility of designing and synthesizing a COF with a specific function. Hence, a wide range of chemicals and biomolecules can be selectively detected by COFs or COF-based hybrid materials. Secondly, the porous structure of the COFs greatly benefits the enrichment of the analyte, significantly enhancing the sensitivity of the detection. Furthermore, the binding site that can selectively interact with the analyte can be easily linked to the COFs. It will remarkably enhance the selectivity of the sensing. Thus, it can be seen that COFs are good candidates for realizing highly sensitive and selective sensing. Thirdly, diverse aromatic structures endow the COFs with rich optical and electrical properties. Moreover, the unique nature of the COFs allows the COFs to play multiple roles in the chemical and biological sensors, including an analyte concentrator, signal generator, and functional particles’ carrier. It will significantly decrease the complexity of the sensing devices.

The above advantages exhibited that COFs are a class of promising materials for chemical and biological sensing. Although great progress has been made, COF-based sensors are still under investigation in the laboratory. However, there are still some remaining disadvantages that inhibit the COF-based sensors from practical application. They will be the important challenges to promoting the utilization of the COF-based sensors. The first challenge is the mass production of the COFs. The monomers for preparing functional COFs need to be obtained by organic synthesis. The complex synthetic route may significantly decrease the yield of the final products, and enhance the cost of COFs production. Furthermore, the method to control the polymerization of the monomers and the crystallization process during the COFs formation is still far from perfect. Obtaining COFs with uniform quality is still difficult to guarantee. Secondly, the most widely used technique for COFs synthesis is the solvothermal method. Therefore, most COFs are obtained as a powder. However, for sensor fabrication, the thin film can exhibit much better performance than the powder due to its uniformity and compactness. Therefore, effective approaches to preparing COFs thin films on various substrates are still eagerly expected. Thirdly, with the development of wearable and implantable sensing devices, the demand for materials with good biocompatibility is increasing. However, the data on the biocompatibility of reported COFs are still deficient. It may limit the further application of COFs in biosensing. COF materials with biocompatibility and biosafety should be developed for promoting its application in domestic, wearable, and implantable sensing devices.

In recent years, substantial efforts have been devoted to creating new COFs with various functions. The increasing number of new COFs will continue to promote its application in the development of chemical and biological sensors. Although the COF-based sensors have been utilized for detecting various analytes, it can still be expected that the emergence of new COFs can further extend the range of the analytes. The selectivity and sensitivity will be further improved. Moreover, with the growing demand for fast and real-time detection of pollutants, dangerous articles, and physiological indexes. Portable, wearable, or even implantable sensors is drawing increasing attention. As mentioned above, COFs are usually able to play multiple roles in a sensor, for example, being an analyte concentrator and signal generator simultaneously. It can remarkably reduce the size and complexity of the sensor. Therefore, COFs also display great potential in constructing miniaturized sensing devices. Additionally, it is also promising to develop COF-based sensors which are suitable to work in special environments, such as the polar region, space environment, and body fluids. Thanks to the increasing eagerness for environmental protection, security, food safety, and healthcare, developing new COF-based chemical or biological sensors with better performance and new applications is still far from the end.

## Figures and Tables

**Table 1:** The dynamic linkages for COF synthesis.

Name	Monomer 1	Monomer 2	Linker
Boronate	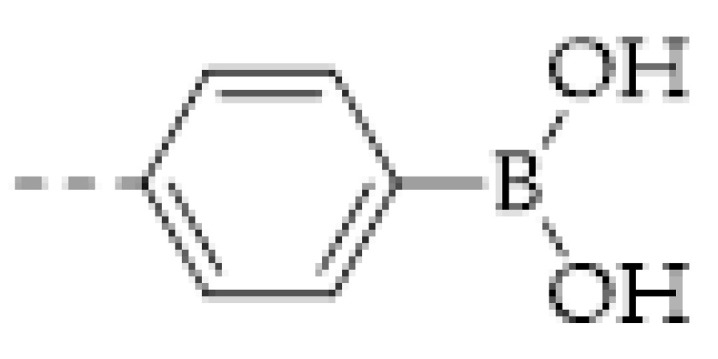	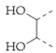	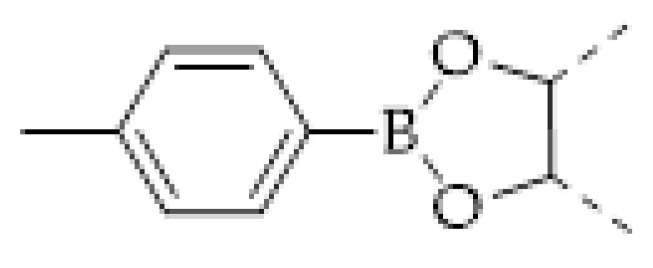
Imine	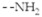	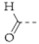	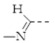
Hydrazone	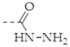	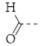	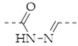
Maleimide	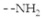	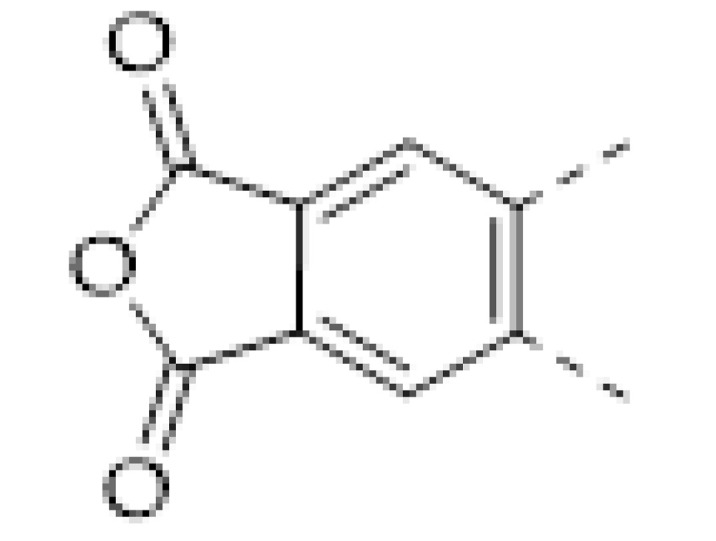	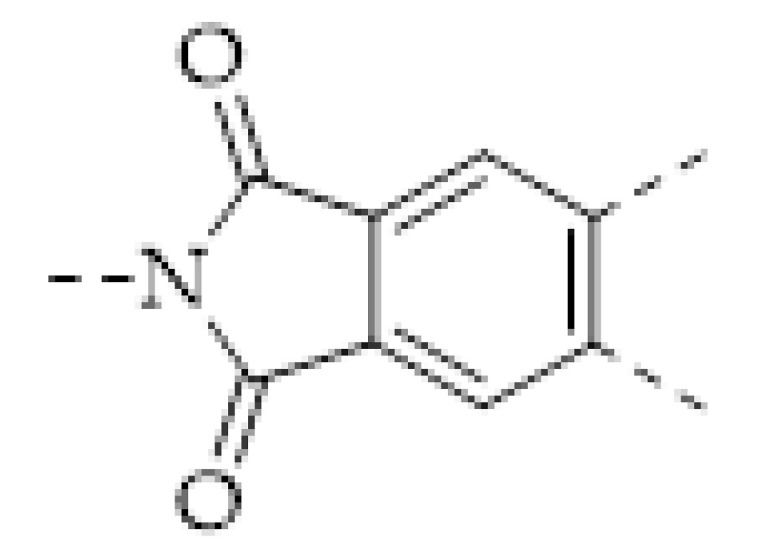
Phenazine	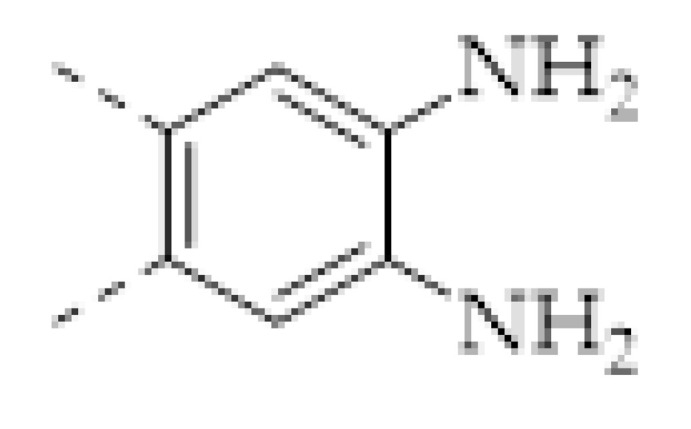	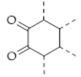	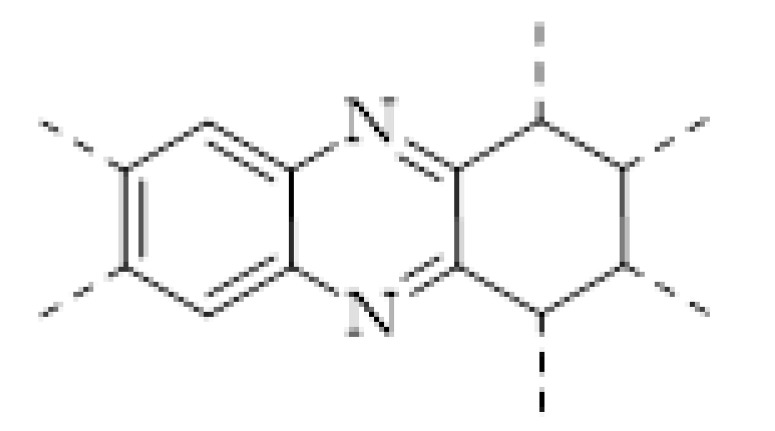
Acrylonitrile	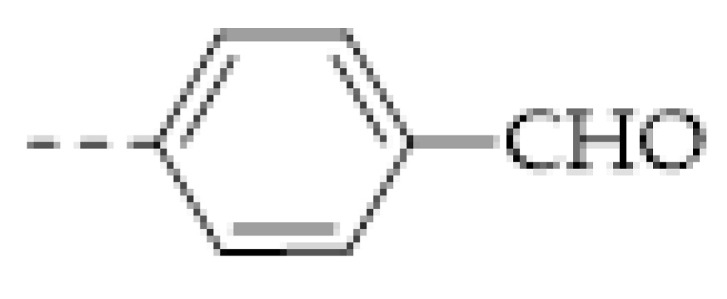	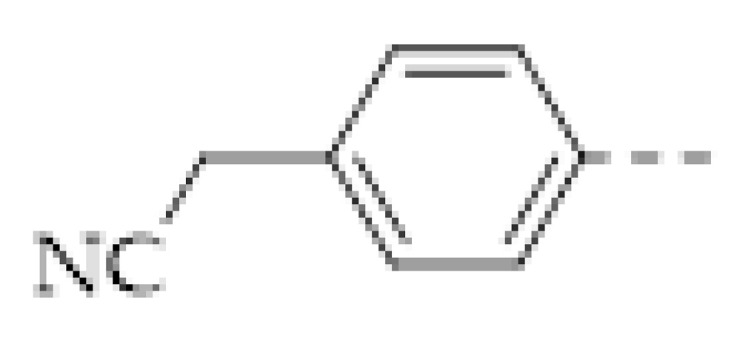	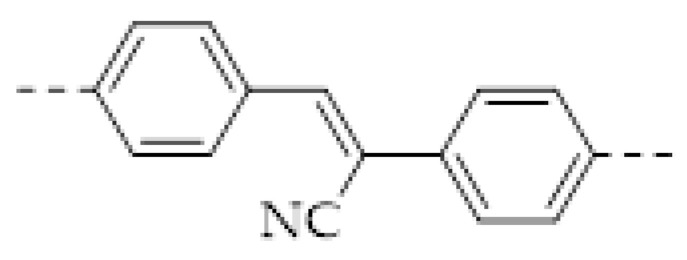
Triazine	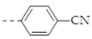		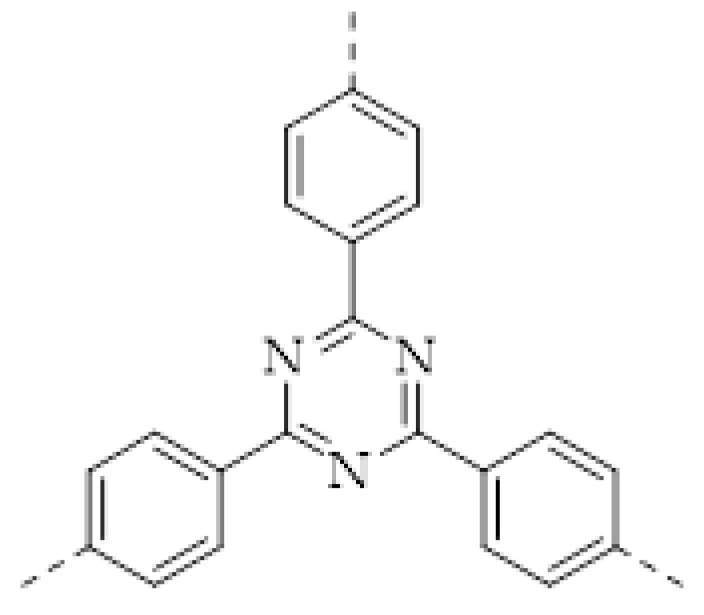
Borazine	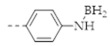		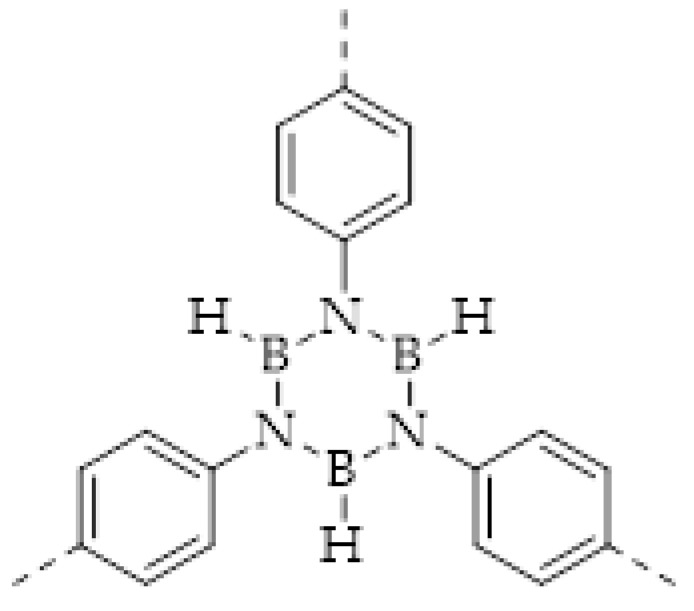
Benzimidazole	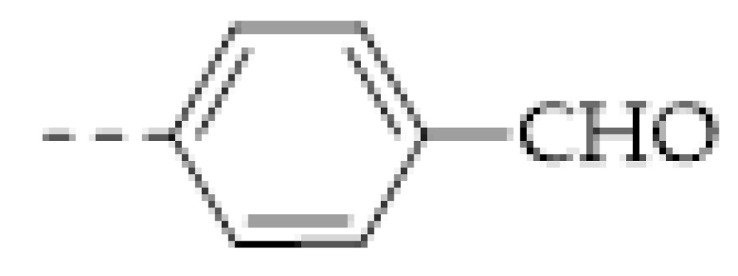	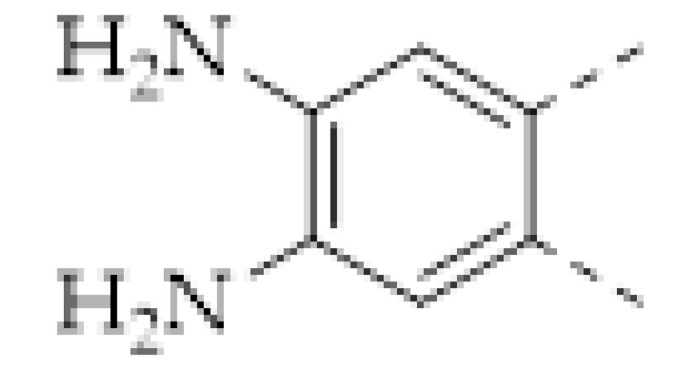	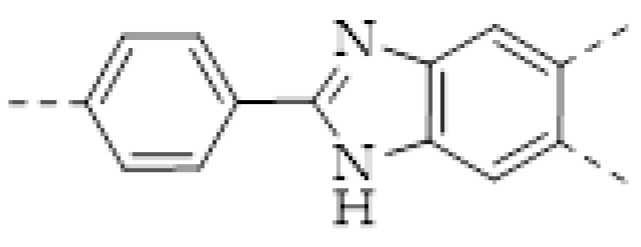
Benzobisoxazole	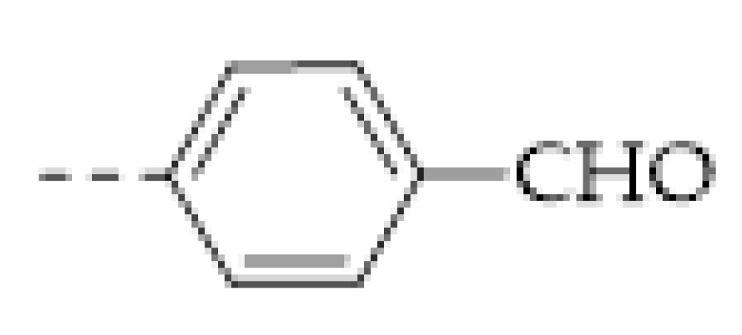	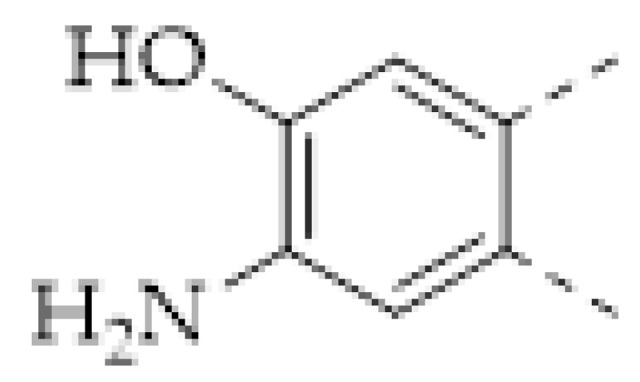	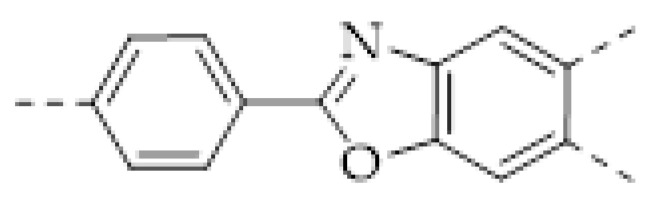

**Table 3:** COF-based ion sensing.

Analyte	Year	COF Names	Specific Binding Site	Type of Detectable Signal	Detection Range	LOD	Reference
Hg^2+^	2016	COF−LZU8	Thioether	Fluorescence, “turn off”	-	25.0 ppb	[42]
	2019	Tp−Bpy NSs	AuNPs	Chromism	-	0.33 nM	[29]
	2020	TFPPy−CHYD	Carbohydrazide	Fluorescence, “turn off”	0.05 μM–4 μM	17 nM.	[73]
	2021	BATHz−Bt	Carbon-carbon double bonds	Fluorescence, “turn off”	0–27.5 mM	26 nM	[74]
Cu^2+^	2016	COF−JLU3	Hydroxyl and azine	Fluorescence, “turn off”	0–0.4 mM	0.31 mM	[75]
	2017	CTF	Triazine	Chromism	1.0 g/L-80.0 g/L	0.05 g/L	[50]
	2017	LMOP−15LMOP−15	Tertiary amino group	Fluorescence, “turn off”		5.1 × 10^−8^ M	[76]
	2018	sp2c-COFs	Cyano groups	Fluorescence, “turn off”		88 ppb	[77]
	2019	QG−scaffolded COF	N atoms and hydroxyl groups	Fluorescence, “turn off”	0.0010~10.0 μM	0.50 nM	[78]
Fe^3+^	2017	PI−COF 201	Amino groups	Fluorescence, “turn off”	5.0–400 μM	0.13 μM	[79]
		PI−COF 202			5.0–300 μM	0.22 μM	
	2019	COF−TT	Amino groups	Fluorescence, “turn off”	0–1.2 mM	0.369 mM	[80]
	2019	TaDAP TaDA	Imine	Fluorescence, “turn off”	0.02–0.2 mM	18 μM	[81]
	2019	Bth−Dha, Bth−Dma	O,N,O′-chelating sites	Fluorescence, “turn off”	-	0.17 μM	[82]
	2021	TTPE−COF		Fluorescence “turn off”	10^−8^–10^−2^ M	3.07 μM	[83]
	2021	Tfpa−Mth COF	Hydrazide and phenol ether	Fluorescence, “turn off” and QCM	-	64 nM	[84]
	2021	PMDA−TAPB	Carbonyl group	Fluorescence, “turn off”	-	-	[85]
Pb^2+^	2018	TAPB-DMTP−COF	Amino groups	Electrochemical signals	0.0050–2.0 μM	0.0019 μmol/L	[86]
	2019	Sulfhydryl modified TAPB−DMTTAPB−DMTTAPB−DMTP−COF	Sulfhydryl	Electrochemical signals	0.05–20 ng·mL^−1^	0.015 ng·mL^−1^	[87]
	2021	TAPP−COF		Photoelectrochemical signal	0.05–1000 nM	0.012 nM	[88]
	2021	PMDA−TAPB	Amino groups	Electrochemical signals	5–9000 nM	1.22 nM	[85]
Au^+^	2018	TTB−COF	Thioether	Fluorescence, “turn on”	1.0–10.0 mM	1.39 mM	[89]
UO_2_^2+^	2020	TFPT−BTAN−AO	Carbon-carbon double bonds	Fluorescence, “turn off”	-	6.7 nM	[90]
	2021	Tph−BDP	Imines of the CT complex	Chromism	0.18–75 μM	0.05 μM	[91]
Ni^2+^	2021	BPD−COFs	N atoms	Fluorescence, “turn off”	0.420–1.26 × 10^3^ pM	68.0 pM	[92]
Cr^3+^	2021	CoPc−PT−COF@Cu−MOF	Bipyridine	Electrochemical signals	10^−1^–10^5^ pM	0.0229 pM	[93]
Pd^2+^	2021	XB−COFs	Carbon-carbon double bonds	Fluorescence, “turn off”	-	0.29 μM	[94]
	2021	PY−SE−COF	Selenodiazole	Fluorescence, “turn off”	20–450 mM	0.45 mM	[95]

**Table 4:** COF-based pH sensing.

Analyte	Year	COF/COF Hybrid Names	Specific Binding Site	Type of Detectable Signal	Detection Range	Reference
H^+^	2016	COF−JLU4	Amine	Fluorescence “turn off”	pH 0.9–13.0	[100]
	2018	COF−HQ	Quinoline	Fluorescence “turn off”	pH 1.0–5.0	[101]
	2019	COF_DHTA-TTA_	-	Electrochemical signals	pH 3.0–11.0	[102]
	2021	COF_2_	Imine or triazine	Fluorescence “turn on”	pH 5.0–8.0	[103]
	2021	COF−TP	Amine	Fluorescence “turn off”	pH 0–6.0	[104]

**Table 5:** COF-based anion sensing.

Analyte	Year	COF Names	Specific Binding Site	Type of Detectable Signal	Detection Range	LOD	Reference
CrO_4_^2−^(Cr_2_O_7_^2−^)	2019	COF−TT		Fluorescence, “turn off”		0.343 mM	[80]
MnO_4_^−^	2019	COF−TT		Fluorescence, “turn off”		0.320 mM	[80]
F^−^	2015	BCMP−3	Boron sites	Fluorescence, “turn off”			[105]
	2018	TFPPy−DETHz−COF	Amine	Fluorescence, “turn off”	-	50.5 ppb	[106]
	2018	2D−Fe−CTF	Triazine	Chromism	10–100 μM	0.56 μM	[107]
S^2^^−^	2018	TpASH	Azide	Fluorescence, “turn on”	1 μM–5 mM	0.12 μM	[108]

**Table 6:** COF-based explosive sensing.

COF Names	Year	Analyte	Type of Detectable Signal	Detection Range	LOD	Reference
SNW−1	2012	picric acid	Fluorescence, “turn off”	0.2–52.4 μM	0.05 μM	[111]
COP−2 COP−3 COP−4	2012	picric acid	Fluorescence, “turn off”	-	~1 ppm	[117]
		TNT			~1 ppm	
Py−Azine COF	2013	picric acid	Fluorescence, “turn off”	0–70 ppm	-	[116]
COF−301 COF−401	2015	picric acid	Fluorescence, “turn off”	-	1 ppm	[112]
iPrTAPB−TFP	2015	picric acid	Fluorescence, “turn off”	-	1 ppm	[118]
TfpBDH−CONs	2015	picric acid	Fluorescence, “turn on/off”	-	1 × 10^−3^ M	[119]
TRIPTA	2016	picric acid	Fluorescence, “turn off”	-	51.96 nM	[120]
3D−Py−COF	2016	picric acid	Fluorescence, “turn off”	0−20 ppm	-	[121]
3′PD	2017	picric acid	Fluorescence, “turn off”			[115]
		triacetone triperoxide				
PI−CONs	2017	picric acid	Fluorescence, “turn off”	0.5–10 μM	0.25 μM	[122]
COP−612 COP−616	2017	picric acid	Fluorescence, “turn off”	-	15 ppm	[113]
LMOP−15	2017	picric acid	Fluorescence, “turn off”	-	0.33 μM	[76]
iPrTAPB−Azo−COP	2018	picric acid	Fluorescence, “turn off”	-	13 ppm	[123]
1	2018	picric acid	Fluorescence, “turn off”	-	68 ppb	[124]
CMP−LS1 CMP−LS2	2018	picric acid	Fluorescence, “turn off”	-	-	[125]
Py−TPE−COF	2018	picric acid	Fluorescence, “turn off”	-	10 ppm	[126]
DL−COF	2019	picric acid	Fluorescence, “turn off”	-	13.10 ppb	[127]
		2,4-dinitrophenol			8.56 ppb	
		2,4-dinitrotoluene			10.40 ppb	
		4-nitrophenol			5.15 ppb	
		4-nitrotoluene			6.92 ppb	
LPCMP1−4	2019	TNT	Fluorescence, “turn off”	0–100 ppm	-	[128]
ANCOF	2020	Dichloran	Fluorescence, “turn off”	-	142 ppb	[129]
		4-nitroaniline		-	89 ppb	
A−COF	2021	picric acid	Fluorescence, “turn off”	-	0.09 μM	[114]
TFPB−TTA COF	2022	DNP	Fluorescence, “turn off”	50 nM–10 μM	18 nM	[130]
		picric acid		50 nM–12.5 μM	16 nM	

**Table 7:** COF-based detection of drugs, insecticides and spices.

Year	COF/COF Hybrid Names	Analyte	Specific Binding Site	Type of Detectable Signal	Detection Range	LOD	Reference
2018	TAPB−DMTTAPB−DMTP−COFs/AuNPs	Chlorogenic acid	-	Electrochemical signals (CV)	1.0 × 10^−8^–4.0 × 10^−5^ mol L^−1^	9.5 × 10^−9^ mol L^−1^	[140]
2018	2D Fe−CTFs	Sarcosine	Sarcosine oxidase	Chromism	10–100 μM	0.56μM	[107]
		ochratoxin A	Aptamer		0.2–0.8 μM	-	
2019	Py−M−COF	Enrofloxacin	Aptamer	Electrochemical signals (EIS)	0.01 pg mL^−1^–2 ng mL−1	6.07 fg mL^−1^	[49]
		Ampicillin			0.001–1000 pg mL−1	0.04 fg mL^−1^	
2019	QD−grafted COFs	Ferulic Acid	Molecular imprinting, amino groups	Fluorescence, “turn on”	0.03–60 mg kg^−1^	5 μg kg^−1^	[141]
2019	MIP/MoS_2_/NH_2_−MWCNT@COF	Sulfamerazine	Molecular imprinting	Electrochemical signals	0.30–2.0 × 10^2^ μM	0.11 μM	[142]
2019	Ce−MOF@MCA	Oxytetracycline	Aptamer	Electrochemical signal (EIS)	0.1–0.5 ng mL^−1^	17.4 fg/mL	[143]
2019	Zr−amide−Por-based 2D COF	Tetracycline	Molecular imprinting	Electrochemical signals	5–60 pM	2.3 pM	[144]
2019	Eu@TpPa−1	Levofloxacin	Europium ions	Fluorescence, “turn off”	10^−6^–10^−2^ M	0.2 μM	[145]
2019	MIOP based on QDs−grafted COFs	Tyramine	H-bond, shape selectivity	Fluorescence, “turn on”	35–35,000 µg/kg	7.0 µg/kg	[146]
				SPE–HPLC	20–2000 µg/kg	5.0 µg/kg	
2019	NUS−30	L-dopa	Azine	Fluorescence, “turn off”			[147]
2019	PATP@AuNPs−crosslinked MIP	Dopamine	Molecular imprinting	electrochemiluminescence	10^–14^−10^−6^ M	2 × 10^−15^ M	[148]
2020	UiO−66−NH2/MCA/MWCNT@rGONR	Kanamycin	Aptamer	Electrochemical signals	25–900 nM	13 nM	[149]
2020	TpPa−1@Dye	Sialic acid	Cr^3+^	Fluorescence, “turn on”	10^−8^–10^−2^ M	7.08 × 10^−9^ M	[150]
2021	Au@COF/GO−NH2	Chloramphenicol	Aptamer	Electrochemical signal (EIS)	0.0001–1 ng mL-1	16.13 fg mL^−1^	[151]
2021	Mg@Fe−MIL−101/TpPa−1−COF	Tetracycline	Mg^2+^	Fluorescence, “turn off”	-	-	[152]
2021	COF−1 or COF−2	Tetracycline	-	Fluorescence, “turn off”	0.005−0.0625 mM	0.002 mM	[153]
		ofloxacin			0.025−0.25 mM	0.0065 mM	
2021	Eu@TpPa−1	5-Fluorouracil	π−π stacking interactions	Fluorescence	10^−7^–10^−3^ M	6.45 × 10^−8^ M	[154]
2018	DAAQ−TFP	Diflubenzuron	Amino and carbonyl group (H bonding) π−π stacking interactions	HPLC	0.2–160.0 ng mL^−1^	0.02 ng mL^−1^	[155]
		Triflumuron			0.2–160.0 ng mL^−1^	0.02 ng mL^−1^	
		Hexaflumuron			0.2–160.0 ng mL^−1^	0.05 ng mL^−1^	
		Teflubenzuron			0.2–160.0 ng mL^−1^	0.04 ng mL^−1^	
2019	NH_2_@COF	Carboxylic acid pesticides	Amino group	HPLC-DAD	0.2–100 ng mL−1	0.04–0.20 ng mL^−1^	[156]
2019	CNs−grafted COFs@MIP	4-ethylguaiacol	The surface of the silica matrix by acid–base pairing interactions	Fluorescence, “turn off”	0.025–1μg ml^−1^	17 ng mL^−1^	[157]

**Table 8:** COFs or COF-based hybrid materials for detecting glucose, uric acids, GSH, and other small biomolecules.

Analyte	Year	COF/COF Hybrid Names	Specific Binding Site	Type of Detectable Signal	Detection Range	LOD	Reference
Glucose	2018	Fe−COF	Glucose oxidase	Chromism	5–350 μM	1.1 μM	[160]
	2019	COF_DHTA-TTA_	Glucose oxidase	Electrochemical signal	0.60 μM–6.0 mM	0.38 μM	[102]
	2020	Fe−PorCOF	Glucose oxidase	Chemiluminescence	0.01–10 μM	5.3 nM	[161]
	2021	COFHD–GOX	Glucose oxidase	Chromism	5–2000 μM	0.54 μM	[162]
Uric acid	2021	COF−DC−8	Hydroxyl, triazine	Electrochemical signals	5.0–25 μM, 25–250 μM	0.77 μM	[163]
Ascorbic acid	2021	COF−DC−8	Hydroxyl, triazine	Electrochemical signals	30–180 μM, 0.18–1.5 μM	12.0 μM	[163]
Dopamine	2021	COF−DC−8	Hydroxyl, triazine	Electrochemical signals	1.0–6.0 μM, 8.0–50 μM	0.25 μM	[163]
GSH	2020	COF−300−AR		Chromism	1–15 μM	1.0 μM	[26]
	2021	Py−TT COF		Chromism	0.4 − 60 μM	0.225 μM	[27]

## Data Availability

No new data were created or analyzed in this study. Data sharing is not applicable to this article.
